# TcSERPIN, an inhibitor that interacts with cocoa defense proteins and has biotechnological potential against human pathogens

**DOI:** 10.3389/fpls.2024.1337750

**Published:** 2024-01-29

**Authors:** Monaliza Macêdo Ferreira, Keilane Silva Farias, Maria Zugaib, Akyla Maria Martins Alves, Geiseane Velozo Amaral, Maria Luíza do Carmo Santos, Andria dos Santos Freitas, Brenda Conceição Guimarães Santana, Sérgio Liberato dos Santos Júnior, Irma Yuliana Mora-Ocampo, Ariana Silva Santos, Marcelo Fernandes da Silva, Bruno Silva Andrade, Carlos Priminho Pirovani

**Affiliations:** ^1^ Centro de Biotecnologia e Genética (CBG), Departamento de Ciências Biológicas, Universidade Estadual de Santa Cruz (UESC), Ilhéus, Bahia, Brazil; ^2^ Laboratório de Bioinformática e Química Computacional (LBQC), Departamento de Ciências Biológicas, Universidade Estadual do Sudoeste da Bahia (UESB), Jequié, Bahia, Brazil

**Keywords:** serpin, *Theobroma cacao*, protease inhibitors, thermostability, stress and defense

## Abstract

In plants, serpins are a superfamily of serine and cysteine protease inhibitors involved in stress and defense mechanisms, with potential for controlling agricultural pests, making them important biotechnological tools. The objective of this study was to characterize a serpin from *Theobroma cacao*, called TcSERPIN, to identify its endogenous targets and determine its function and biotechnological potential. TcSERPIN has 390 amino acid residues and shows conservation of the main active site, RCL. Cis-elements related to light, stress, hormones, anaerobic induction, cell cycle regulation and defense have been identified in the gene’s regulatory region. TcSERPIN transcripts are accumulated in different tissues of *Theobroma cacao*. Furthermore, in plants infected with *Moniliophtora perniciosa* and *Phytophthora palmivora*, the expression of TcSERPIN was positively regulated. The protein spectrum, rTcSERPIN, reveals a typical β-sheet pattern and is thermostable at pH 8, but loses its structure with temperature increases above 66°C at pH 7. At the molar ratios of 0.65 and 0.49, rTcSERPIN inhibited 55 and 28% of the activity of papain from *Carica papaya* and trypsin from *Sus scrofa*, respectively. The protease trap containing immobilized rTcSERPIN captured endogenous defense proteins from cocoa extracts that are related to metabolic pathways, stress and defense. The evaluation of the biotechnological potential against geohelminth larvae showed that rTcSERPIN and rTcCYS4 (*Theobroma cacao* cystatin 4) reduced the movement of larvae after 24 hours. The results of this work show that TcSERPIN has ideal biochemical characteristics for biotechnological applications, as well as potential for studies of resistance to phytopathogens of agricultural crops.

## 1 Introduction

Protein inhibitors are present in different species and are proteins basically responsible for controlling the activity of endogenous proteases (Kim et al., 2009; Bonturi et al., 2022), playing important roles in the regulation of many biological processes (Grosse-Holz and van der Hoorn, 2016). In plants, many of these inhibitors act as storage proteins (Ostergaard et al., 2000; Grosse-Holz and van der Hoorn, 2016), control important endogenous proteases during seed germination (Grosse-Holz and van der Hoorn, 2016; Tanner et al., 2019) and modulate plant tolerance to various stresses (Benchabane et al., 2010; Koh et al., 2016; Martinez et al., 2016; Lema Asqui et al., 2018). In addition, many of these inhibitors have biotechnological potential against phytopathogens or agricultural pests, making them potential targets for agronomic studies (Alvarez-Alfageme et al., 2011; do Amaral et al., 2022).

Among the most prominent protease inhibitors, serpins constitute a superfamily of protein inhibitors with broad inhibitory capacity against different serine and cysteine proteases, and are involved in plant defense mechanisms. Although the name “serpin” is derived from the initial observation that these proteins were characterized as inhibitors of serine proteases (Dahl et al., 1996a; Dahl et al., 1996b; Ostergaard et al., 2000; Gettins, 2002; Ostergaard et al., 2004), it is now known that this superfamily of inhibitors can also inhibit proteases of the cysteine class (Lampl et al., 2013; Rustgi et al., 2017; Lema Asqui et al., 2018). In addition, some serpins identified have no inhibitory potential and are related to other functions that are still poorly understood (Cohen and Fluhr, 2018; Tolstyko et al., 2021).

Serpins are highly conserved proteins and have a unique structural pattern, typical of the superfamily, with 7 – 9 α-helices, 3 β-sheets and a reactive central loop (RCL), which is where the interaction with target proteases occurs (Irving et al., 2000).

Due to their mechanism of action, serpins are known as suicide substrate inhibitors. Basically, during the interaction mechanism, the protease recognizes the RCL of the serpin as a potential substrate and cleaves the scissile bond between residues P_1_ – P_1’_ (Irving et al., 2000), according to the nomenclature established by Schechter and Berger (1967) for the substrate of proteases, in which the cleavage of the bond is between residues P_1_ and P_1’_ (Schechter and Berger, 1967). After cleavage of the loop, a covalent bond is formed between the cleaved loop and the catalytic site of the protease, and the C-terminal end of the serpin is displaced. The residues of the loop are sequentially inserted into the β A-sheet of the serpin, and the protease is dragged to the opposite pole of the inhibitor (on average 70 Å). As a result, the enzyme suffers structural distortion of the catalytic site and is unable to hydrolyze the covalent bond, remaining bound to the serpin (Huntington et al., 2000; Gettins, 2002; Cohen et al., 2019).

In plants, serpins from barley, wheat, pumpkin and thale cress (*Arabidopsis thaliana*) have been characterized and their inhibitory potential revealed (Dahl et al., 1996a; Dahl et al., 1996b; Ostergaard et al., 2000; Yoo et al., 2000; Lampl et al., 2010). Serpins such as AtSerpin1 (Lampl et al., 2013; Chen and Fluhr, 2018; Lema Asqui et al., 2018), proteinase inhibitor I4 (MtPiI4) and serpin 6 (MtSer6) from *Medicago truncatula* (Sun et al., 2015; Dhanushkodi et al., 2018), and serpin LRS (OsSRP-LRS) from *Oryza sativa* (Francis et al., 2012; Bhattacharjee et al., 2015) are among the widely studied examples. These have been suggested as proteins acting in the regulation of proteolytic activity and control of cell death caused by biotic and abiotic stresses, with biotechnological potential against insect pests (Alvarez-Alfageme et al., 2011).

Given the inhibitory capacity of serpins and their role in plant defense mechanisms, a serpin was identified in the cacao tree, genotype Belizian Criollo B97-61/B2 (Argout et al., 2011), named TcSERPIN. In order to validate the role of TcSERPIN in *T. cacao*, we carried out *in silico*, *in vitro* and *in vivo* studies to characterize the protein and determine its function, identify possible targets in the leaf protein extract and establish its pathways in cellular mechanisms. Our results revealed that rTcSERPIN has a thermostability profile at pH 8, and apparently interacts with an endogenous cysteine protease, and that the expression of the serpin gene increases when cocoa is infected by the pathogens *Moniliophthora perniciosa* and *Phytophthora palmivora*, highlighting its relationship with the plant defense. Furthermore, rTcSERPIN showed inhibitory potential against the movement of geohelminth larvae, which are nematodes found in the soil that cause diseases in humans.

## 2 Materials and methods

### 2.1 Analysis of the TcSERPIN gene and the amino acid residues corresponding to the protein

The complete sequence of the cocoa TcSERPIN gene and protein (access code Tc08v2_p001150.1) was obtained from the Belizian Criollo B97-61/B2 genome, available in the Cocoa Genome Hub (https://cocoa-genome-hub.southgreen.fr/) (Argout et al., 2017). After identifying the gene, a cis-element analysis was performed using 1500 bp upstream of the 5’UTR of the coding region. The presence of cis-regulatory elements was analyzed using the plantCARE server (sphinx.rug.ac.be:8080/PlantCARE/cgi/index.html) (Lescot et al., 2002).

Possible post-translational modifications in the amino acid residues of the protein, such as the presence of a signal peptide, putative sites of phosphorylation, glycosylation, acetylation and subcellular localization, were evaluated. For these analyses, the following programs were used: SignalP 4.0 Server (http://www.cbs.dtu.dk/services/SignalP-4.0/) (Petersen et al., 2011); NetPhos 2.0 (http://www.cbs.dtu.dk/services/NetPhos-2.0/) (Blom et al., 1999); NetNGlyc 1.0 Server (http://www.cbs.dtu.dk/services/NetNGlyc/) (Gupta and Brunak, 2002); NetAcet 1.0 Server (http://www.cbs.dtu.dk/services/NetAcet-1.0/) (Kiemer et al., 2005); and TargetP (http://www.cbs.dtu.dk/services/TargetP/) (Emanuelsson et al., 2007), respectively.

To compare conserved sites between cacao serpin and other homologous sequences, we performed alignment using the Clustal Omega tool (https://www.ebi.ac.uk/Tools/msa/clustalo/) (Sievers et al., 2011). The sequences of all serpins used in the alignment were obtained through searches using the Basic Local Alignment Search Tool (BLAST) (https://blast.ncbi.nlm.nih.gov/Blast.cgi) (Altschul et al., 1997), using TcSERPIN as bait.

### 2.2 Transcriptional profile of cocoa inhibitory proteins

The transcriptional profile of inhibitors (TcSERPIN and TcCYS4) was evaluated for different cacao organs and biotic stress.

To carry out the analyses, a cacao Criollo genome file in fast format (GCA_000208745.2_Criollo_cocoa_genome_V2/) was downloaded from the GenBank database (https://www.ncbi.nlm.nih.gov/genbank/) and used as a reference file for the RNA-Seq libraries (https://www.ncbi.nlm.nih.gov/sra) of leaves, flowers, buds (SRP148703), pistils (SRP004925), and seeds (SRP136974), as well as plants infected by the pathogens *M. perniciosa* (SRA066232), and *P. palmivora* (SRP248100). All the libraries used were obtained from public data available from NCBI SRA (https://www.ncbi.nlm.nih.gov/sra). Subsequently, the transcripts per million (TPM) values were calculated by the RNA Galaxy workbench 2.0 software (https://rna.usegalaxy.eu), using the Salmon extension, a method that quantifies the abundance of transcripts from RNA-seq reads (Patro et al., 2017).

The RNA-Seq libraries referring to plant-pathogen interaction represent replicates of control conditions (healthy plants) and infected plants. To determine transcript accumulation levels under biotic stresses, a heat map was generated using the R Studio software and Complex Heatmap packages, based on normalized data and Euclidean distance.

The identity between the transcripts and the corresponding proteins were analyzed using the BLAST platform (https://blast.ncbi.nlm.nih.gov/Blast.cgi).

### 2.3 Obtaining plant material

Leaves (Scavina 6 genotype) were collected from adult plants, with age of 5 years, available in the CEPLAC germplasm bank in field conditions (Executive Committee of the Cacao Plantation Plan), Ilhéus, Bahia (14°45’40. 2”S 39°14’03. 9”W), under registration number A8AD1C0 in SisGen (National System for Management of Genetic Heritage and Associated Traditional Knowledge), authorizing collection through access to genetic heritage.

### 2.4 Expression and purification of recombinant TcSERPIN

For *in vitro* functional testing, the synthetic rTcSERPIN clone (Tc08v2_p001150.1) was obtained from the company Biomatik (Kitchener, Ontario, Canada) following the cloning strategy in pET28a, containing the restriction sites for *NcoI/XhoI*. *Escherichia coli* strain *Rosetta (DE_3_)* was transformed with the recombinant plasmid by the heat shock method (Sambrook and Russell, 2001), and the transformed colonies were selected on LB (Luria-Bertani) medium and agar containing kanamycin (50 μg/mL) and chloramphenicol (50 μg/mL). The induction of the recombinant protein was carried out in the Circle Grow medium containing the antibiotics, at 37°C, under stirring at 180 rpm until reaching OD 600 nm between 0.7 – 1.0, then incubated with 0.4 mM of IPTG (isopropyl-β-D-thiogalactopyranoside) at 18°C for 16 h, under stirring. After the induction period, the bacterial extract was centrifuged at 15500 g for 20 minutes.

Recombinant serpin was purified from soluble and insoluble fractions, according to Alves et al. (2019), with modifications. Both fractions were loaded into TALON^®^ Superflow™ cobalt-based resin (GE Healthcare), following the manufacturer’s instructions, and eluted with an elution buffer containing 150 mM imidazole (soluble fraction) and 6 M urea (insoluble fraction).

rTcSERPIN purified from the soluble fraction was dialyzed in 10 mM Tris HCL and 1X phosphate buffered saline (PBS) (137 mM NaCl, 10 mM Na_2_HPO_4_, 2mM KH_2_PO_4_, 2.7 mM KCl, pH 7.4), which were the buffers that caused greatest stability of the recombinant protein during dialysis. For the insoluble fraction, the protein was subjected to gradual refolding and reduction of urea concentration in the dialysis buffer (10 mM Tris HCL). The previously characterized inhibitor (Pirovani et al., 2010), rTcCYS4 (*Theobroma cacao* cystatin 4), was expressed and purified from the soluble fraction of the total bacterial extract according to Pirovani et al. (2010).

The result of the induction and purification of recombinant proteins was verified by sodium dodecyl sulfate-polyacrylamide gel electrophoresis (SDS-PAGE) at 12.5%, as described by (Laemmli, 1970), and stained with Coomassie Blue G250 (Neuhoff et al., 1988). The concentration of purified proteins was determined by the Bradford method (Bradford, 1976), in a SpectraMax microplate reader (Molecular Devices) using bovine serum albumin (BSA) to calculate the standard curve.

### 2.5 Analysis of the secondary structure and thermal stability of rTcSERPIN by circular dichroism (CD)

The thermal stability of rTcSERPIN was analyzed by circular dichroism spectroscopy (CD) with a Jasco J-815 spectropolarimeter equipped with a Peltier PTC-423S/15 temperature control. A total of 200 μg/mL of the protein dialyzed in Tris HCL and citrate buffers was placed in a 1 mm quartz cuvette, and the stability of the secondary structure of rTcSERPIN was evaluated at basic pH (10 mM Tris HCL pH 7 and 8) and acid pH (10 mM citrate pH 4 and 6). The choice of buffers was based on observation of the greater stability of the protein during the dialysis. The spectra were read using the Spectra Measurement software (Jasco) at temperatures of 25 °C and 95 °C, wavelengths of 190 – 240 nm, scan rate of 50 nm.minute^-1^ and intervals of 0.5 nm, for data collection. An average of six scans were performed at each reading.

The analyses of unfolding (25 to 95 °C) and refolding (95 to 25 °C) were carried out by measuring changes in the secondary structure of the protein, in 10 mM Tris HCL, pH 7.0, at a wavelength of 216 nm. All unfolding and refolding analyses were performed in triplicate and the average was used to calculate the denaturation percentage of the secondary structure under varying temperature and pH conditions.

### 2.6 Evaluation of the inhibitory activity of cocoa serpin

The *in vitro* inhibitory activity of the serpin was determined according to the optimum conditions for the activity of proteases (papain and trypsin), which were determined previously (Pirovani et al., 2010; do Amaral et al., 2022), with adaptations. Against *Carica papaya* papain, the analyses were carried out in 50 mM phosphate buffer, pH 6.0, with 10 mM β-mercaptoethanol and 2 mM EDTA. For *Sus scrofa* trypsin, the assay was performed in 50 mM Tris-HCL buffer, pH 7.4, and 20 mM CaCl_2_. All analyses were carried out at 30 °C using the chromogenic substrate Nα-benzoyl-DL-arginine-4-nitroanilide hydrochloride, BApNA (1.2 mM).

Determination of the inhibitory activity of rTcSERPIN was performed using different molar concentrations of serpin against 0.01067 µmol of papain and 0.00840 µmol of trypsin (**Supplementary Table 1**). The residual activity of the proteases was measured by substrate hydrolysis (BApNA) and monitored in a VersaMax microplate spectrophotometer (Molecular Devices), with reading at 410 nm and intervals of 5 min, for 30 min. The inhibition percentage of papain and trypsin by rTcSERPIN was calculated according to Pirovani et al. (2010), and the inhibitor-protease molar ratio was determined.

### 2.7 Capture of *T. cacao* proteins by rTcSERPIN in a system immobilized by CNBr-Sepharose

For the capture assay, rTcSERPIN and bovine serum albumin (BSA) (negative control) were coupled to CNBr-activated SepharoseTM 4 fast Flow (GE Healthcare) according to the manufacturer’s instructions.

The total leaf extract (genotype Scavina 6) was obtained under non-denaturing conditions, according to Pirovani et al. (2008), using extraction buffer (Tris-HCl 10 mM pH 7.5, Triton X-100 1%), 0.4 volume of tert-butanol and 1/10 volume of sodium acetate 3M, pH 4.5. After extraction, the total leaf extract was incubated with serpin in buffers suitable for serine-type target protease activity (50 mM Tris-HCL buffer, pH 7.4, and 1 mM CaCl2) and cysteine (50 mM phosphate buffer, pH 6.0, 10 mM β-mercaptoethanol and 2 mM EDTA), for 1 h, at room temperature, under agitation. Subsequently, the steps to obtain the proteins that interacted with rTcSERPIN were performed according to Santos et al. (2023).

The activity of the captured proteases was evaluated using 0.4% gelatin/SDS polyacrylamide gel electrophoresis (Michaud et al., 1996), and proteins were identified by mass spectrometry (LC - MS/MS). Papain from *C. papaya* (0.05 µg) was used as positive control, and the eluate resulting from the interaction between BSA and the leaf extract was used as negative control.

### 2.8 Identification of proteins captured by rTcSERPIN using mass spectrometry (LC - MS/MS)

The identification of proteins by mass spectrometry was performed using the total extract of cocoa leaves and the resulting eluate after the interaction between rTcSERPIN together with CNBr-Sepharose resin and the leaf protein extract.

The samples were reduced and alkylated using dithiothreitol (DTT) and iodoacetamide (IAA), respectively, and diluted in 50 mM NH_4_HCO_3_ (1:5) and 1 mM CaCl_2_. The protein solution was digested with trypsin according to the method of Villén and Gygi (2008), with adaptations, and desalted using C18 resin tips (10 µL; Millpore^®^) according to the manufacturer’s recommendations. Peptides were eluted in 50 µL of a solution containing 50% acetonitrile, 25% water and 0.1% formic acid, and analyzed by liquid chromatography with an Agilent 1290 Infinity II UPLC system coupled to a quadrupole/time-of-flight mass spectrometer (Agilent 6545 LC/QTOF).

The generated spectra were processed in triplicate for peptide identification using the Spectrum Mill software (Rev B.06.00.203 SP1; Agilent). The parameters for spectra extraction were: MSNoiseThreshold (10 counts); fixed changes (carbamidomethylation); MH+ precursor (200 to 6000 Da); retention time tolerance (+/- 60 secs); tolerance m/z +/- 1.4; precursor charge (find).

After extracting the MS/MS spectra, a search for proteins was performed in the *T. cacao* database, downloaded from UniProt (https://www.uniprot.org). The parameters used to compare the MS/MS spectra in the protein bank were: maximum number of missed cleavages (= 2); fixed post-translational modifications such as carbamidomethylation of cysteine (C), and variable post-translational modifications defined by oxidation of methionine (M), pyroglutamic acid (N-termQ), deamidation of asparagine (N), and phosphorylation of serine, threonine and tyrosine (S, T, Y). The combined minimum peak intensity was 10%; and the precursor mass tolerance was set at +/- 20 ppm. Search results were validated and filtered for those that had a false positive rate (FDR) of less than 1%, score greater than 5 and scored peak intensity (SPI) greater than 60%. Results were exported in protein-protein comparison mode in MPP APR file format. Proteomic archives (doi:10.25345/C5862BP0X) are available online at the Center for Computational Mass Spectrometry (https://massive.ucsd.edu/ProteoSAFe/dataset.jsp?accession=MSV000093675).

### 2.9 Protein-protein interaction network (PPI)

To understand the interactions between TcSERPIN and endogenous *T. cacao* proteins, an interaction network was created based on homology with *A. thaliana* proteins. The protein sequences used in the network analysis were obtained from direct searches of the UniProt platform (The UniProt Consortium, 2023) (https://www.uniprot.org/).

Protein network analysis was performed in STRING 10.5 (http://string-db.org) (Szklarczyk et al., 2023), according to Mora-Ocampo et al. (2021) with the following parameters: (i) line thickness of network edges as an indication of the level of confidence; (ii) active interaction sources such as text mining evidence, experiments, databases, co-expression, neighborhood, gene fusion and co-occurrence; minimum required interaction score of 0.7 (high confidence); and maximum number of interactions to show 1st and 2nd shells (no more than 50 interactions). The file of each network was downloaded in TSV format and later the files were merged with Cytoscape version 3.6.0. The Igraph package of R Studio was used to calculate the parameters of centrality (betweenness) and nodes (degree). The analysis of the genetic ontology of network clusters, which determines their functions, was carried out in STRING 10.5 (http://string-db.org).

### 2.10 Modeling and docking

Prediction of the three-dimensional (3-D) structures of *T. cacao* proteins (TcSERPIN and cysteine protease) were performed using the online SWISS-MODEL tool (https://swissmodel.expasy.org/) (Waterhouse et al., 2018). As a template to generate the 3-D model of TcSERPIN, the crystal structure of AtSerpin1 (PDB Code: 3LE2) from *A. thaliana* in its native stressed conformation was used. The modeling of the cysteine protease from cocoa was performed only with the sequence of the C1 peptidase domain, which is the mature form of the enzyme, and the prediction of the 3-D model used a cysteine endopeptidase (PDB Code: 1s4v.2) from *Ricinus communis* as template. PROCHECK was used to evaluate the stereochemical quality of 3-D models generated according to Ramachandran graphs (Laskowski et al., 1993).

Molecular docking between the 3-D models of TcSERPIN (receptor) and the cocoa cysteine protease (ligand) was performed using ClusPro 2.0 (http://cluspro.bu.edu/), and the chosen docking model considered estimates of the energy levels (van der Walls, electrostatic and hydrophobic forces), the size of each cluster generated, and the interactions present at the active sites of the proteins involved.

The manipulation and visualization of the 3-D models was performed using PyMol (PyMOL Molecular Graphics System, version 1.5.0.4 Schrödinger, LLC.) and Discovery Studio 4.0 programs.

### 2.11 Studies with the mycelium of *Moniliophthora perniciosa*

The isolate of the fungus *M. perniciosa* (ID number CCMB 257) was provided by the Microorganism Culture Collection of Bahia (CCMB) of Feira de Santana State University (UEFS). The fungus was preserved in Castellani medium for 15 days at 25 °C and reactivated in potato dextrose agar (PDA) medium.

Mycelial discs of the fungus (22 mm in diameter) were inoculated in the center of the Petri dish containing PDA medium together with filter paper discs (20 mm in diameter) soaked in 1X phosphate buffered saline (PBS) pH 7.4 (control) and 1X PBS containing the rTcSERPIN at different concentrations (50, 100, 200 and 300 µg/mL). Paper discs were added to the ends of the plate, and the solutions (control and protein) were replenished every 2 days for 6 days.

### 2.12 Studies with geohelminth larvae

The samples were harvested in the spring and summer seasons only on days with weather forecast of sunshine without rain at Cristo Beach in Ilhéus, Bahia (-14.806034155046431, -39.031712471689765) during low tide, in places with high risk of contamination by geohelminth larvae, as described previously by (Silva et al., 2019). Then, the samples were processed using a protocol based on the traditional (Harada and Mori, 1955) technique to isolate the larvae from beach sand. As a result, the volume containing the larvae was centrifuged at 2,000 g to collect several sediments and reduce the number of samples under analysis. After centrifugation, the number of geohelminth larvae was counted and their motility was checked (typical movement within 15 seconds). According to the morphological aspects observed in the study, the most of the larvae were in the filariform infective stage.

For the test, two recombinant proteins were used: TcSERPIN, the focus of this work, and TcCYS4, a cysteine protease inhibitor characterized by Pirovani et al. (2010), which is highly promising and has demonstrated activity against the mycelial growth of *M. perniciosa*. The recombinant proteins were initially tested against geohelminth larvae at the concentrations obtained immediately after purification and dialysis, at 220 and 130 µg/mL for rTcSERPIN and rTcCYS4, respectively. Subsequently, to evaluate the activity of the inhibitors at higher concentrations, the proteins were added to 15 mL concentrator tubes containing Vivaspin 10 kDa MWCO columns until reaching a maximum concentration at which there was no protein precipitation. rTcSERPIN was concentrated to a maximum value of 330 µg/mL. So that the two inhibitors could be compared, rTcCYS4 was used at a concentration of 320 µg/mL. To compare the effects of recombinant proteins on larval mobility, 1X PBS buffer (137 mM NaCl, 10 mM Na_2_HPO_4_, 2mM KH_2_PO_4_, 2.7 mM KCl, pH 7.4), water and formaldehyde (2%) were utilized as controls. 1X PBS and water were the negative control and the biocide formaldehyde was used as a positive control (Santos et al., 2012).

In order to evaluate of the potential of the recombinant proteins and the controls on geohelminth motility, the larvae were placed in Petri dishes with the protein solution for 24 h, at room temperature. The experiment was carried out in triplicate, and involved a suspension of larvae. The sample number for the parameter analyzed varied between 6 and 8 larvae. Larval motility (tail movement in 15 seconds) in the presence of the treatment solutions (proteins) and the controls (water, PBS 1X and formaldehyde) was assessed using an optical microscope (400x magnification), and the readings of the presence or absence of larval movement were checked by three people. The mean and standard deviation were calculated for the treatments, and the quantitative data on larval immobility were submitted to analysis of variance (ANOVA) and the Tukey test using the Sisvar software with p-value < 0.05.

## 3 Results

### 3.1 Analysis of the genic and regulatory regions of the *TcSERPIN* gene

**Figure 1:**
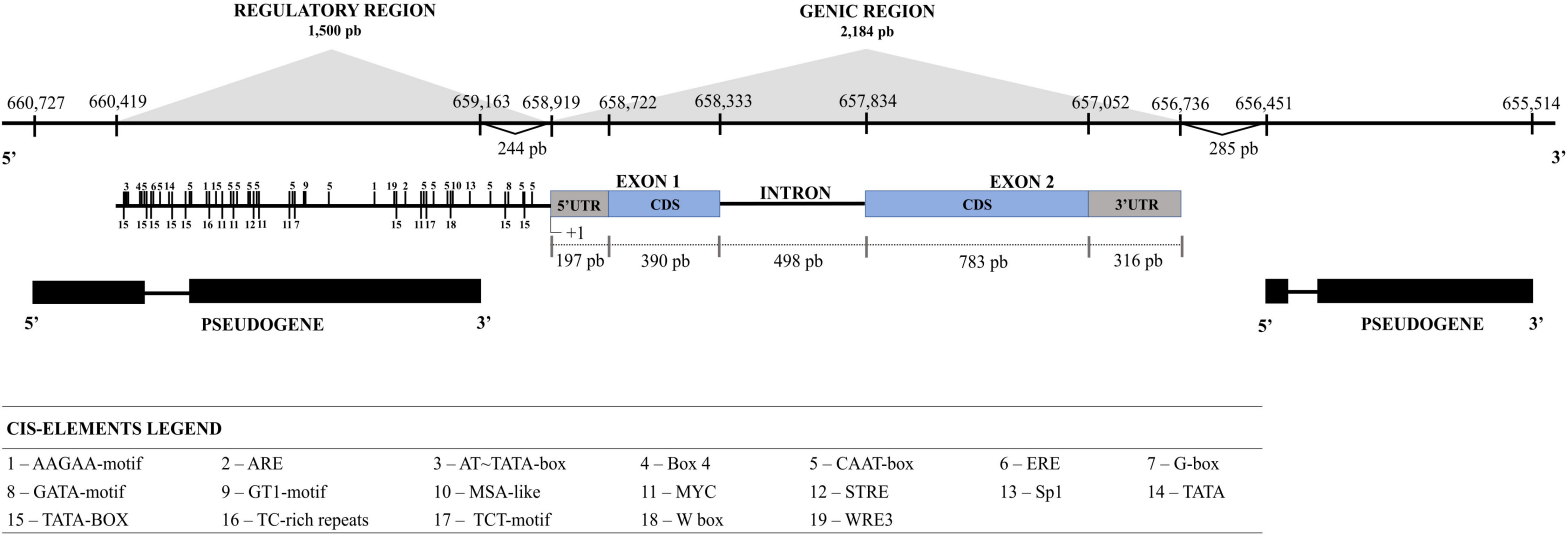
Analysis of the gene and regulatory regions of *TcSERPIN*. The size of the gene and the regulatory region in bp is shown on the scale. Coding regions are shown in gray and blue. The intron and regulatory region (1500 bp upstream of the 5’UTR of the gene) is represented by the black line. Pseudogenes are shown in black immediately below the *TcSERPIN* gene. The cis-elements are represented by numbers 1500 bp from the gene.

Based on research at the Cocoa Genome Hub, the serpin gene was identified on chromosome 8 and named *TcSERPIN*. The gene region is 2,184 bp, with 2 exons and 1 intron, and is flanked by two serpin pseudogenes 244 and 285 bp upstream of the 5’ and 3’ UTR regions, respectively (**Figure 1**). Sequence alignment between genes showed that *TcSERPIN* and the two pseudogenes had 76.9 and 65.6% identity, respectively (**Supplementary Figure 1**).

Analysis of the regulatory region 1,500 bp upstream of the 5’ UTR (coding region) showed several conserved cis-elements. Among the identified cis-elements were AT~TATA-box, CAAT-box, TATA and TATA-box, which are enhancer elements and responsive to transcription initiation. In addition, cis-elements related to luminosity (Box 4, G-box, GATA-motif, GT1-motif, Sp1, TCT-motif), defense and stress (ARE, MYC, STRE, TC-rich repeats, W Box, WRE3), ethylene and abscisic acid hormone pathways (ERE and MYC, respectively), and cell cycle regulation (MSA-like) were identified (**Figure 1**; **Supplementary Table 2**).

### 3.2 TcSERPIN amino acid sequence analysis

The serpin ORF with 1,173 pb was found to encode a predicted protein of 42.5 kDa with a putative pI of 5.88. TcSERPIN had 390 amino acid residues, 20 putative phosphorylation sites and two glycosylation sites (**Supplementary Figure 2**). No signal peptide was detected.

**Figure 2:**
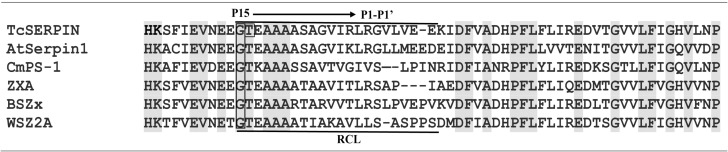
Excerpt from the alignment of the TcSERPIN protein and homologues. The alignment presents the section that contains the amino acid residues that are present in the RCL (reactive center loop) of the serpins of *T. cacao* (TcSERPIN, Tc02v2_p008580.1), *A. thaliana* (AtSerpin1, NP_190108.1), *C. maxima* (CmPS-1, AAG02411.1), *O. sativa* (ZXA, XP_015632921.2), *H. vulgare* (BSZx, Q40066.1), *T. aestivum* (SPZ2A, Q9ST57.1). Conserved regions are highlighted in gray. The main region of the RCL, P_15_ (glycine) to P_1’_ (arginine), is highlighted in the image. Gap regions are indicated by (–). The phosphorylation site is indicated in a rectangle.

The comparative alignment between the serpin domain of TcSERPIN, CmPS-1 (*Curcubita maxima*), AtSerpin1 (*A. thaliana*), BSZx (*Hordeum vulgare*), SPZ2A (*Triticum aestivum*) and ZXA (*Oryza sativa*) showed that the proteins share identity above 50% and conservation of the major active site, the RCL, marked by the presence of amino acid residues such as glycine (P_15_), threonine (P_14_) and alanine (P_12_ - P_10_) in all serpins (**Figure 2**; **Supplementary Figure 2**). Furthermore, the serpins TcSERPIN, AtSerpin1, ZXA and BSZx displayed the leucine-arginine pair in the P_1_ – P_1’_ region, a probable site of cleavage by cognate proteases (**Figure 2**).

### 3.3 Accumulation levels of cacao serpin transcripts

**Figure 3:**
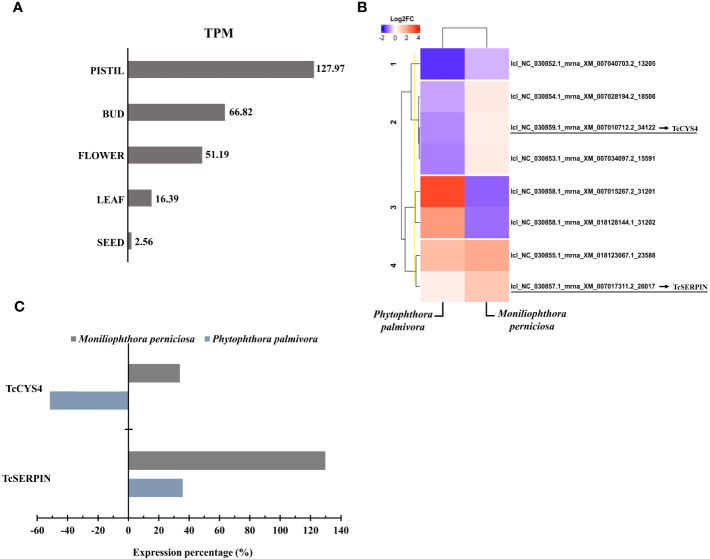
Transcriptional profile of cocoa inhibitors. **(A)** Relative quantification of *TcSERPIN* transcripts accumulated in different parts of the cocoa plant. The TPM (transcripts per million) values of the lcl_NC_030857.1_mrna_XM_007017311.2_26017 transcript are shown in the image. **(B)** Heat map according to Pearson’s correlation showing the differential expression of transcripts corresponding to *TcSERPIN* and *T. cacao* cystatins in plants infected by *Phytophthora palmivora* and *Moniliophtora perniciosa* compared to uninfected plants. The transcripts identified in the reference file of the cacao Criollo genotype for the cacao inhibitors are underlined in the image. The blue and red colors classify the expression level of the negatively and positively regulated transcripts, respectively, according to the Log2FC scale of -10 and 5. **(C)** Percentage of expression of *TcSERPIN* and TcCYS4 transcripts in cocoa plants infected by the pathogens *M. perniciosa* (gray) and *P. palmivora* (blue) compared to healthy plants.

The TcSERPIN transcript, lcl_NC_030857.1_mrna_XM_007017311.2_26017, were expressed in different cocoa organs, and according to Blastx, it had 100% identity with the protein (XP_007017373.2). According to TPM values, TcSERPIN transcripts were more expressed in pistils (TPM: 127.97), followed by flower buds (TPM: 66.82), flowers (TPM: 51.19), leaves (TPM: 16.39) and seeds (TPM: 2.56) (**Figure 3A**). TcSERPIN was almost 50 times more expressed in pistils (**Figure 3A**).

Of the 34,771 transcripts quantified, TcSERPIN ranked 1,406th in number of transcripts per gene in pistils, 2,928th in flowers, 2,856th in floral buds, 7,397th in leaves, and 17,715th in seeds (**Supplementary Table 3**).

**Figure 3B** shows the transcriptional profile of the transcripts corresponding to the TcSERPIN and TcCYS4 proteins in response to the biotic stresses caused by the fungus *M. perniciosa* and the oomycete *P. palmivora*. A total of 7 cystatin transcripts were identified in the reference file, and the transcript lcl_NC_030859.1_mrna_XM_007010712.2_34122, with 98.54% identity, refers to the TcCYS4 protein (**Figure 3B**).

The transcriptional profile of *TcSERPIN* and *TcCYS4* genes was higher when the plant was infected by *M. perniciosa*, with increases of 130 and 34% in expression compared to the control, respectively. In contrast, against the oomycete *P. palmivora*, TcSERPIN transcripts only increased by 36%, while the expression of the TcCYS4 transcripts was 52% lower (**Figure 3C**).

### 3.4 Induction and purification of recombinant proteins

SDS-PAGE analysis of bacterial extracts containing the recombinant proteins TcSERPIN and TcCYS4 induced by IPTG revealed the presence of His-tagged proteins, with molecular weights of approximately 45 and 27 kDa, respectively. Both proteins were expressed and purified from soluble fractions. rTcSERPIN, however, was also purified from the insoluble fraction of the bacterial extract, since the protein had a high concentration in this fraction, as can be observed by the SDS-PAGE result (**Supplementary Figure 3**).

### 3.5 Secondary structure analysis of rTcSERPIN

**Figure 4:**
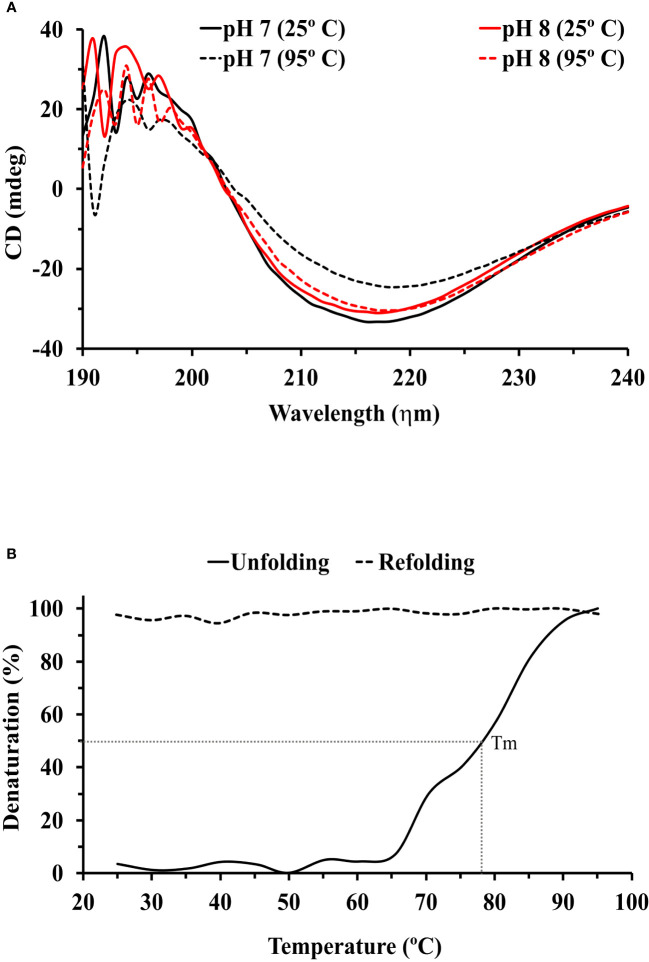
Circular dichroism (CD) analysis of rTcSERPIN. **(A)** Spectral profiles by circular dichroism at wavelengths from 190 to 240, at pH 7 and 8. The black and red lines correspond to the spectra at pH 7 and 8, respectively. The solid lines refer to the reading of the spectra at 25°C, and the dashed lines at 95°C. **(B)** Unfolding (25 to 95°C) and refolding (95 to 25°C) of rTcSERPIN at 216 nm, pH 7. The unfolding analysis is represented by a solid line and refolding by a dashed line. The gray dotted line shows the Tm of the protein at 78 °C. The analysis was performed with the average of three independent readings from each treatment.

The circular dichroism (CD) spectrum of the purified rTcSERPIN protein of the *E. coli* extracts showed positive peaks at the absorbances of 196 nm (28.87 mdeg) and 197 nm (28.35 mdeg), and two negative peaks at the absorbances of 216 (-33.5 mdeg) and 217 nm (-31.17 mdeg) at pH 7 and 8, respectively (**Figure 4A**).

At pH 7, the spectrum generated at 25 °C (lowest signal at -33.5 mdeg) did not overlap with the spectrum at 95 °C (lowest signal at -24.55 mdeg), showing variation at the two contrasting temperatures (25 and 95° C). However, at pH 8, the smallest signals presented by the spectra at 25 and 95 °C (-31.17 and -30.39 mdeg) overlapped without showing significant changes (**Figure 4A**).

Evaluation of unfolding and refolding was performed at pH 7, the condition of greatest variation in the protein spectrum. rTcSERPIN maintained stability up to 66°C, with Tm of 78 °C. From this temperature onwards, the protein gradually denatured and did not refold when subjected to analysis at 95 to 25 °C (**Figure 4B**). We also observed that precipitation of rTcSERPIN occurred, visible in the cuvette at the end of the analysis and absent when the temperature increase occurred at pH 8 (**Supplementary Figure 4A**).

Analyses of the rTcSERPIN spectrum at pH below 7, using citrate buffer (10 mM), showed a similar signal to that exhibited at 95°C and pH 7, in which the protein was denatured and therefore was not used in the final analysis (**Supplementary Figures 4B, C**).

### 3.6 Inhibitory activity of rTcSERPIN against trypsin from *S. scrofa* and papain from *C. papaya*

**Figure 5:**
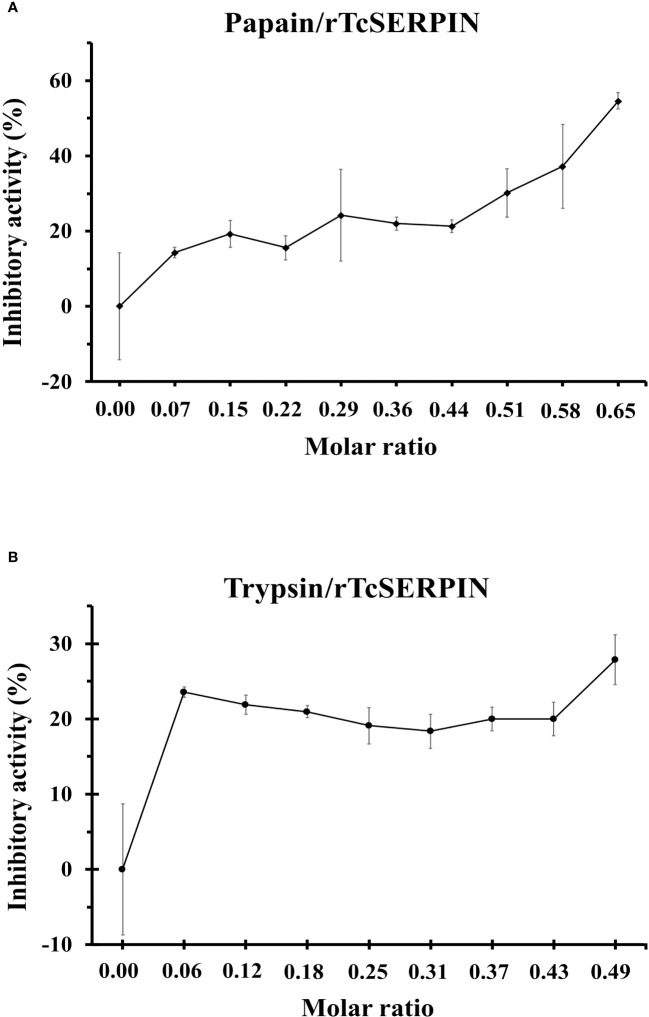
rTcSERPIN inhibitory activity. **(A, B)** Analysis of the inhibitory activity of rTcSERPIN against papain from *Carica papaya* and trypsin from *Sus scrofa*, respectively. The inhibitory test was performed at different molar concentrations of the purified inhibitor (rTcSERPIN) and 0.001067 and 0.000840 µmols of papain and trypsin, respectively (**Supplementary Table 1**). Readings were taken at 410 nm for 30 minutes. Variations in the residual activity of proteases are presented as standard errors of the means (*n = 5*).

*In vitro* inhibitory analysis of rTcSERPIN against serine and cysteine proteases revealed that rTcSERPIN exhibited greater inhibition against papain, a cysteine protease. At a molar ratio of 0.65, rTcSERPIN inhibited 55% of papain activity (**Figure 5A**). In turn, the inhibitory percentage of rTcSERPIN against *S. scrofa* trypsin showed less effect, reaching a maximum value of 28%, with a molar ratio of 0.49 (**Figure 5B**).

### 3.7 Capture of cysteine proteases from *T. cacao* leaf by rTcSERPIN

**Figure 6:**
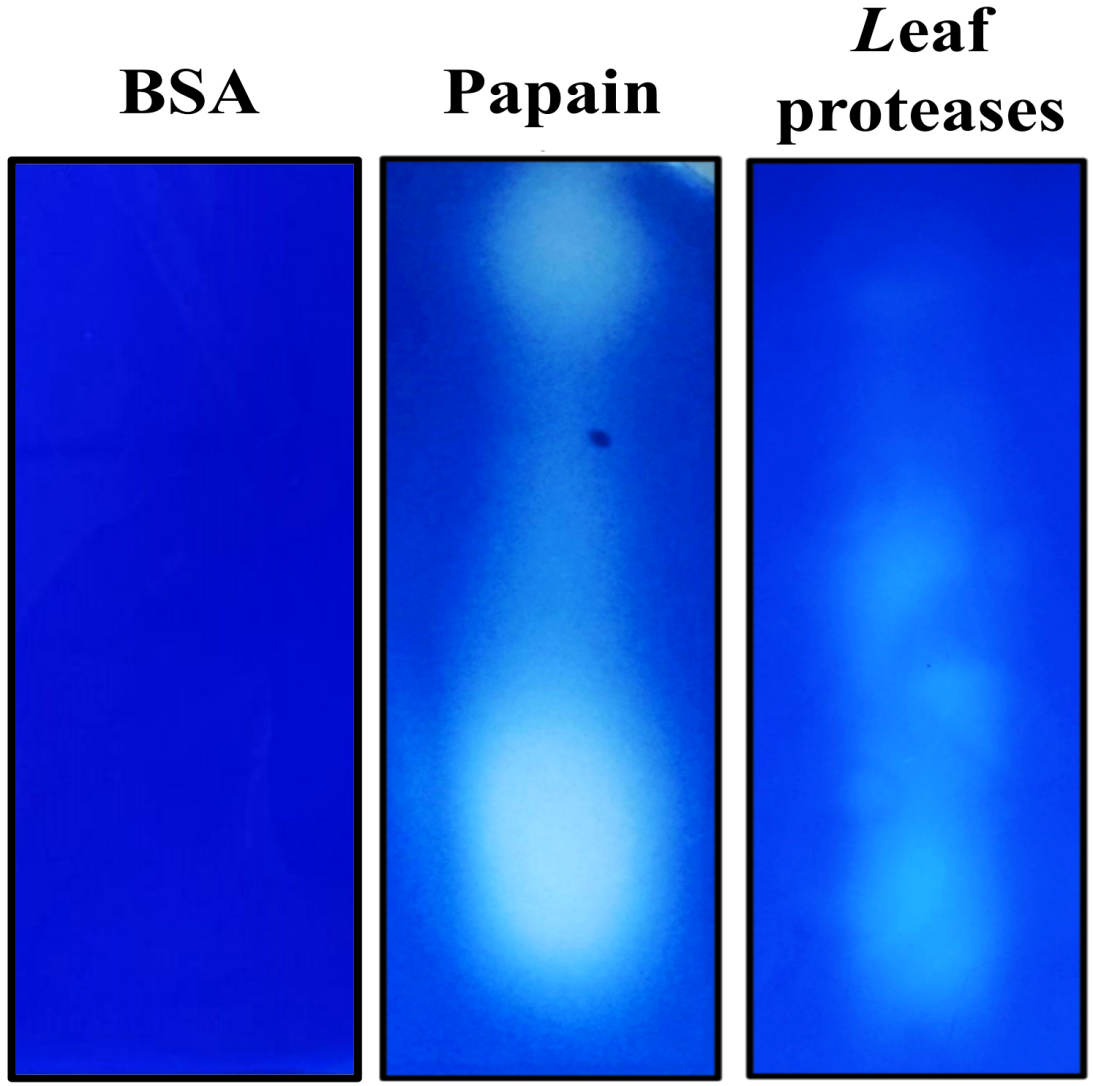
Capture analysis of *Theobroma cacao* leaf proteases by rTcSERPIN. The capture result was analyzed in gelatin/SDS-PAGE. The image shows the activity of cocoa proteases captured and eluted after interaction with rTcSERPIN immobilized on CNBr-activated Sepharose and BSA (negative control). As a positive control, 0.05 µg of papain from papaya was applied in the electrophoresis gel.

The eluate resulting from the capture of proteins from cocoa leaf extract using rTcSERPIN as bait in CNBr–Sepharose showed protease activity, as revealed by the clear halo present on Gelatin/SDS-PAGE. As a comparison, the positive control (papain) also showed a clear halo, while the negative control obtained from the BSA eluate did not have captured protease activity (**Figure 6**).

The profile observed in gelatin/SDS-PAGE showed that rTcSERPIN can interact with endogenous proteases, which can be strong candidates for *in vivo* interaction. Using the eluate from the coupling, a total of four proteins were identified by LC-MS/MS in treatments with Tris HCL or phosphate buffers: 21 kDa seed protein, granulin repeat cysteine protease family protein, galactosyltransferase 13, tetratricopeptide repeat (TPR)-like superfamily protein and thioredoxin-dependent peroxiredoxin (**Table 1**).

**Table 1:** Proteins captured from the total extract of *T. cacao* leaf by rTcSERPIN immobilized in CNBr-activated sepharose.

Phosphate buffer, β-mercaptoethanol and EDTA*								
No. of Spectra^a^	Distinct peptides^b^	Distinct summed MS/MS search score^c^	% AA coverage^d^	Total protein spectral intensity^e^	Protein MW^f^	Protein pI^g^	Database accession^h^	Protein name
18	5	72.36	29.2	2.47e+06	24278.9	5.71	A0A061FWL5	21 kDa seed protein
2	1	12.68	6.3	2.00e+05	24438.0	5.70	A0A061G2K6	21 kDa seed protein
3	1	12.87	4.2	1.12e+05	52613.9	5.09	A0A061FBU0	Granulin repeat cysteine protease family protein
3	1	9.11	1	3.29e+05	65790.8	9.31	A0A061EK43	Galactosyltransferase 13, putative
1	1	5.65	2.5	1.24e+05	84628.7	9.05	A0A061FHV8	Tetratricopeptide repeat (TPR)-like superfamily protein, putative
1	1	11.69	5.2	3.09e+04	29447.1	7.83	A0A061EVI6	Thioredoxin-dependent peroxiredoxin
Tris-HCL and CaCl_2_*								
No. of Spectra^a^	Distinct peptides^b^	Distinct summed MS/MS search score^c^	% AA coverage^d^	Total protein spectral intensity^e^	Protein MW^f^	Protein pI^g^	Database accession^h^	Protein name
10	3	35.87	22.8	4.20e+05	24278.9	5.71	A0A061FWL5	21 kDa seed protein
2	1	12.67	5.8	3.03e+04	24438.0	5.70	A0A061G2K6	21 kDa seed protein
2	1	12.63	4.2	4.10e+04	52613.9	5.09	A0A061FBU0	Granulin repeat cysteine protease family protein

* Total leaf protein extract was incubated with serpin immobilized on CNBr-Sepharose resin in two different buffers for protease activity: 50 mM Tris-HCL, pH 7.4, and 1 mM CaCl_2_; and 50 mM phosphate, pH 6.0, 10 mM β-mercaptoethanol and 2 mM EDTA.

a Number of spectra corresponding to the identified peptides of each protein.

b Number of identified distinct peptides of the corresponding protein.

c Total score for all the distinct peptides in a subgroup. Only proteins with score ≥ 5 were considered.

d Percentage of the protein that was covered by the identified peptides.

e Total intensity of all peptides assigned to that protein. Peptide intensities are calculated from extracted ion chromatograms from the precursor ions.

f Protein molecular weight in Daltons.

g Protein isoelectric point.

h Database: UniProt *Theobroma cacao* proteome.

Among the proteins shown by LC-MS/MS results, the granulin repeat cysteine protease corresponds to a papain homologous to RD21 from *A. thaliana* with 73% identity, as observed by the alignment between the two cysteine proteases (**Supplementary Figure 5**).

Proteins 21 kDa seed and granulin repeat cysteine protease were identified in both treatments with buffers optimized for the activity of serine and cysteine proteases. The other proteins (thioredoxin-dependent peroxiredoxin, galactosyltransferase 13 and tetratricopeptide repeat (TPR)-like superfamily protein) were identified only in the treatment in which the leaf protein extract was treated with the phosphate buffer containing β-mercaptoethanol and EDTA (**Table 1**).

Analysis of the quantitative abundance profile of the proteins resulting from the capture in comparison to the total proteins extracted from the leaf revealed that the 21 kDa seed protein was among the most abundant proteins, while the granulin repeat cysteine protease and the thioredoxin-dependent peroxiredoxin were 8 and 4 times less abundant, respectively, than the 21 kDa seed protein. In addition, galactosyltransferase and the TPR-type protein were not identified, revealing a very low abundance of these proteins, which may have been masked by other more abundant proteins in the sample (**Supplementary Table 4**).

### 3.8 Protein-protein interaction network (PPI)

The PPI network was initially built with the five proteins captured by rTcSERPIN, identified by LC-MS/MS. However, part of these proteins did not form clusters with TcSERPIN, a key criterion to proceed with the study. Therefore, the network was plotted only with the three proteins whose clusters were connected to each other: TcSERPIN; granulin repeat cysteine protease; and thioredoxin-dependent peroxiredoxin.

**Figure 7:**
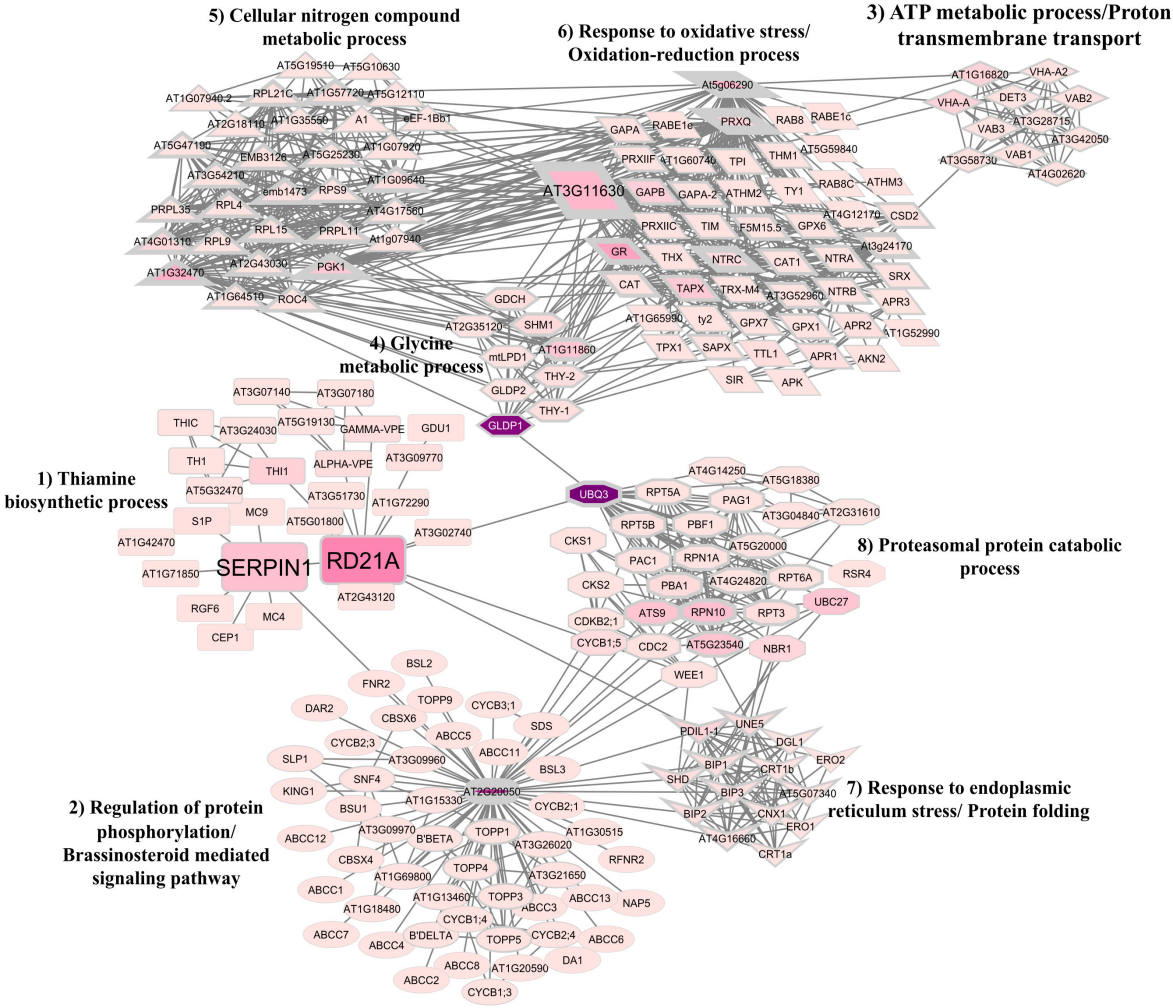
Protein-protein interaction (PPI) network between TcSERPIN and *Theobroma cacao* proteins according to homology with *Arabidopsis thaliana*. Gene ontology analysis revealed the formation of eight clusters that configured the PPI network: Thiamine biosynthetic process (cluster 1); Regulation of protein phosphorylation/Brassinosteroid mediated signaling pathway (cluster 2); ATP metabolic process/Proton transmembrane transport (cluster 3); Glycine metabolic process (cluster 4); Cellular nitrogen compound metabolic process (cluster 5); Response to oxidative stress/Oxidation-reduction process (cluster 6); Response to endoplasmic reticulum stress/Protein folding (cluster 7); and Proteasomal protein catabolic process (cluster 8). Proteins with higher degree values are represented by geometric figures with thicker edges. Proteins with higher betweenness values are represented by geometric figures with darker colors.

The analysis of gene ontology to determine the function of clusters was performed according to *A. thaliana* protein ontology. The PPI network was formed with 218 proteins (nodes) and 1112 connectors (**Figure 7**). Among the proteins found, 29 were considered bottlenecks (betweenness value above average) and 85 were hubs (node degree value above average). In this network, eight clusters were identified: Thiamine biosynthetic process (cluster 1); Regulation of protein phosphorylation/Brassinosteroid mediated signaling pathway (cluster 2); ATP metabolic process/Proton transmembrane transport (cluster 3); Glycine metabolic process (cluster 4); Cellular nitrogen compound metabolic process (cluster 5); Response to oxidative stress/Oxidation-reduction process (cluster 6); Response to endoplasmic reticulum stress/Protein folding (cluster 7); and Proteasomal protein catabolic process (cluster 8) (**Figure 7**).

The three orthologous proteins of the proteins captured by rTcSERPIN were identified as hub and bottleneck proteins and made connections in their own cluster and with other clusters in the network (**Figure 7**). Of these, the cysteine protease represented by RD21 (papain) formed ten connections with proteins (THI1, AT5G01800, AT3G51730, ALPHA-VPE, GAMMA-VPE, AT1G72290, AT3G09770, AT3G02740, AT2G43120, Serpin1) from its own cluster, related to the thiamine biosynthetic process, and three interactions with the proteins UBQ3 and CYCB1; 5, which were related to the proteasomal protein catabolic process (cluster 8), and PDIL1-1 involved in the response to endoplasmic reticulum stress and protein folding (cluster 7) (**Figure 7**).

Serpin1 was also a bottleneck protein and had connections with the main protein of cluster 2 (regulation of protein phosphorylation/brassinosteroid mediated signaling pathway), AT2G20050, and six connections in its own cluster, with the proteins RD21, MC4, MC9, RGF6, AT1G71850 and S1P.

The At3g11630 (thioredoxin-dependent peroxiredoxin) protein had a large number of connections to its own cluster 6, related to response to oxidative stress and the oxidation-reduction process, and was connected with the clusters of ATP metabolic process and proton transmembrane transport (cluster 3), glycine metabolic process (cluster 4), and cellular nitrogen compound metabolic process (cluster 5). At3g11630, however, had direct connection with cluster 1 of serpin and RD21.

### 3.9 Homology modeling and molecular docking between *TcSERPIN* and the granulin repeat cysteine protease family protein from cacao

**Figure 8:**
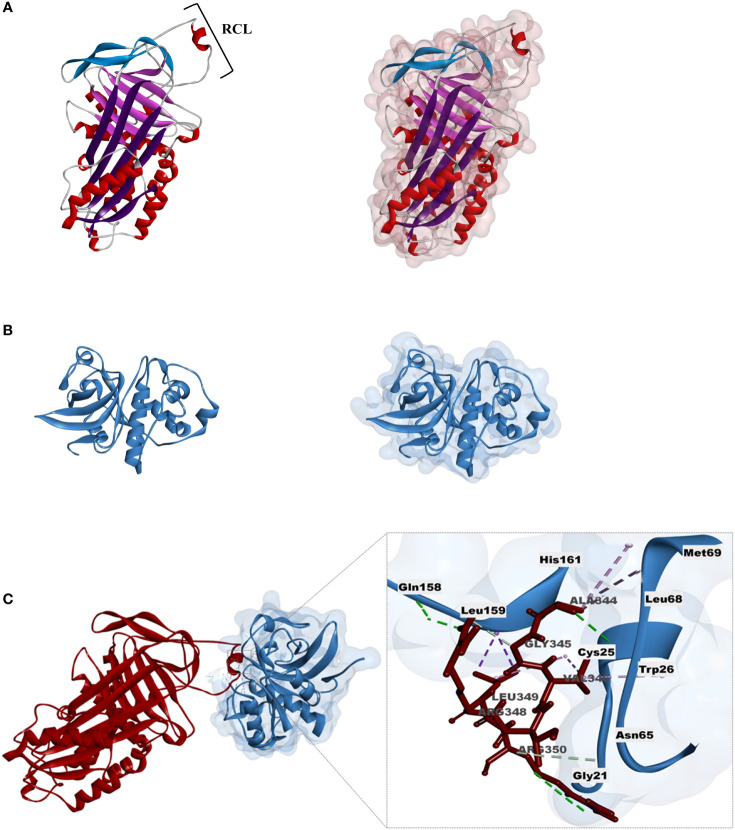
Homology modeling and molecular docking of TcSERPIN and the granulin domain cysteine-protease. **(A)** Three-dimensional structure of TcSERPIN obtained from homology modeling with AtSerpin1 from *Arabidopsis thaliana* (PDB code: 3LE2). In highlight, RCL, the main active site of serpins. On the right, half-surface representation of the inhibitor. **(B)** Three-dimensional structure of the cysteine-protease according to homology with *Ricinus communis* L. (PDB code: 1S4V.2). On the right, half-surface representation of the protease. **(C)** Molecular docking between TcSERPIN and the cysteine-protease. The interaction regions of the serpin RCL (Ala_344_, Gly_345_, Val_346_, Arg_348_, Leu_349_, Arg_350_) and the amino acid residues of the protease (Cys_25_, Gly_21_, Trp_26_, Asn_65_, Leu_68_, Met_69_, Gln_158_, Leu_159_, His_161_) are highlighted in the image.

The 3D model of the TcSERPIN protein was obtained according to homology with the target model AtSerpin1 from *A. thaliana* (PDB Code: 3LE2), with 67.8% identity and 99% coverage. As observed, the 3D structure of TcSERPIN revealed the presence of three β-sheets, nine α-helices and the presence of its main active site, the RCL (**Figure 8A**).

For the 3D structure of the cysteine protease from cocoa, we used the granulin repeat cysteine protease (PDB: 1s4v.2) from *Ricinus communis* as a template, with 99% coverage and 68.5% identity (**Figure 8B**). For the modeling of the enzyme, only the cysteine protease domain was used in its mature form, according to previous results (Guo et al., 2012) (**Supplementary Figure 5**). The validation of the three-dimensional structures was obtained according to the Ramachandram map (**Supplementary Figure 6**), where it was observed that more than 90% of the protein residues were in energetically favorable regions.

Docking was performed to analyze the possible sites of interaction between TcSERPIN and the captured cysteine protease. The analysis resulted in 20 possible conformations, of which we selected only the most favorable complex, according to the selection criteria (see material and methods).

The complex showed energy of -647.3 E/kT. Non-covalent interactions such as hydrogen (H) bonds and hydrophobic interactions were established between the proteins (**Figures 8C**) . The main active site of TcSERPIN, region P_7_ – P_1_ of the RCL (Ala_344_, Gly_345_, Val_346_, Arg_348_, Leu_349_, Arg_350_) interacted with the catalytic triad (Cys_25_ and His_161_), as well as other amino acid residues (Gly_21_, Trp_26_, Asn_65_, Leu_68_, Met_69_, Gln_158_ and Leu_159_) of the cysteine protease (**Figure 8C**).

Interestingly, leucine 349 (P_2_) of TcSERPIN showed alkyl and pi-sigma type hydrophobic interactions with the catalytic residues of the protease, cysteine 25 and histidine 161, respectively. Furthermore, catalytic cysteine was also observed forming an alkyl bond with another RCL residue, valine 346 of the serpin, which in turn also formed a pi-alkyl bond with tryptophan 26 of the protease. Other hydrophobic or hydrogen interactions were also observed between the molecules (**Figure 8C**).

### 3.10 Biotechnological potential of cocoa inhibitors

**Figure 9:**
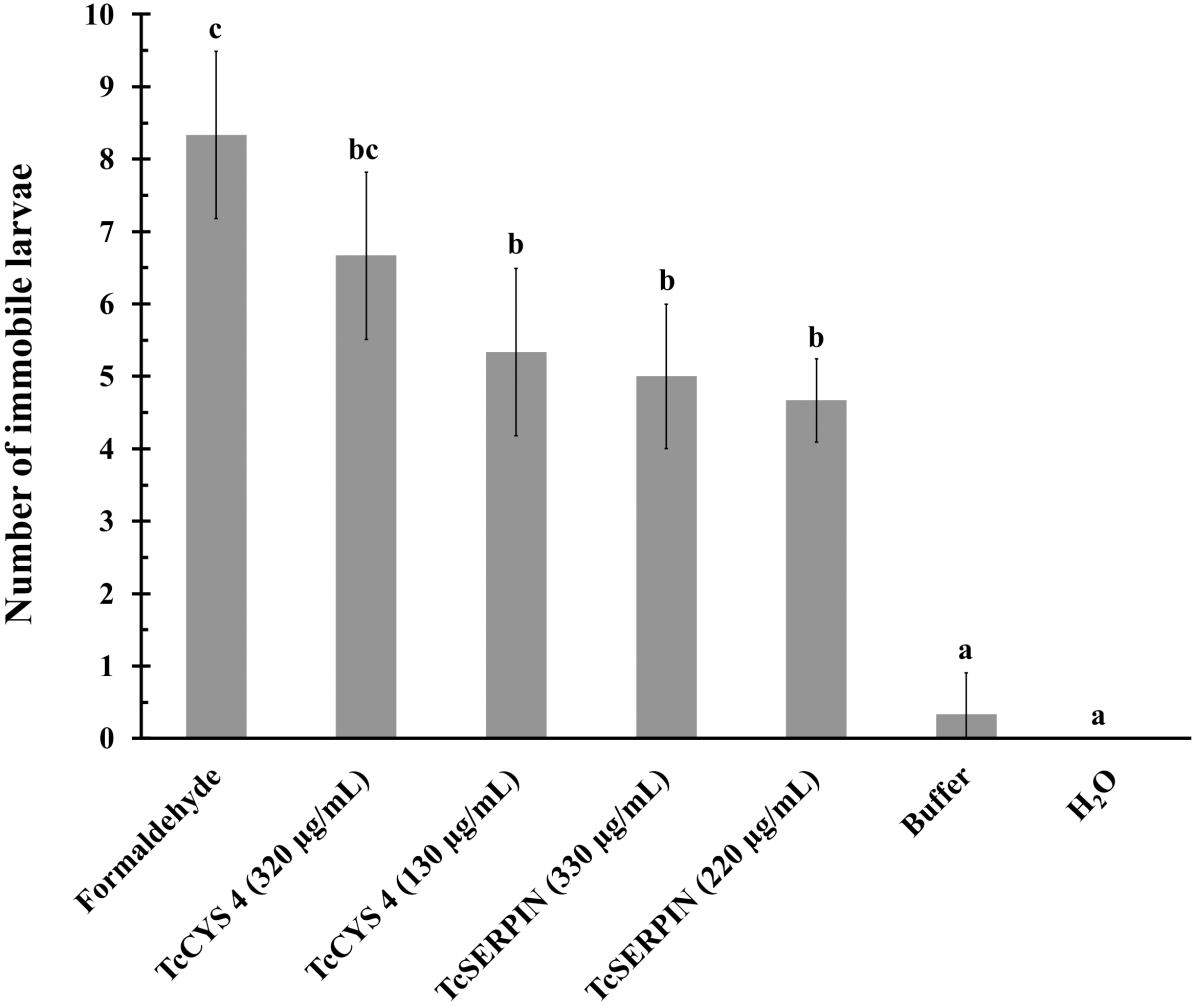
Evaluation of the biotechnological potential of rTcSERPIN and rTcCYS4 against geohelminth larvae. The tests were carried out in triplicate, and the sample size for the analyzed parameter varied between 6 and 8 larvae in each dish. The bars correspond to the standard deviation of the mean. Quantitative data were submitted to analysis of variance (ANOVA) and the Tukey test. Different letters in the columns indicate statistical difference, while identical letters do not differ from each other according to the Tukey test (P<0.05).

Analyses with *M. perniciosa* using rTcSERPIN at different concentrations showed no interference in the mycelial growth of the fungus (**Supplementary Figure 7**). However, the evaluation of the biotechnological potential of rTcSERPIN against geohelminth larvae, which cause cutaneous larva migrans, inhibited larval mobility (**Figure 9**).

As observed in **Figure 9**, there was a statistical difference between the negative controls (H_2_O and 1X PBS) and the recombinant proteins, according to the Tukey test at 5% significance. At the different concentrations tested, both proteins inhibited or reduced larval movement, but there was no statistical difference between them. However, rTcCYS4 at the concentration of 320 µg/mL was similar to the positive control (formaldehyde) (**Figure 9**).

## 4 Discussion

### 4.1 TcSERPIN showed conserved primary structure typical of plant inhibitory serpins

A new serpin was identified in the Criollo cacao genome. This protein has 390 amino acid residues and conservation of the main RCL residues, mainly in the P_15_ - P_1’_ region (**Figure 2**). The conservation of this loop is extremely important for the activity of serpins, as it is the site used to capture the target proteases and carry out their function (Gettins, 2002).

Inhibitory serpins exhibit unique features of the superfamily, such as the presence of a conserved glycine at position P_15_ and 3 to 4 alanines between P_12_ – P_10_, which are short side-chain amino acid residues, important so that the insertion of the loop between the β A-sheet can be fast enough to trap the target protease (Gettins, 2000; Irving et al., 2000). Furthermore, TcSERPIN and the serpins AtSerpin1 from *Arabidopsis*, ZXA from rice and BSZx from barley exhibited an ‘LR’ pair at the P_2_ - P_1_ position (**Figure 2**). It is well known that ‘LR’ serpins are promiscuous inhibitors and have been documented in plant defense and cell death mechanisms (Roberts and Hejgaard, 2008; Cohen et al., 2019). ‘LR’ serpins, such as AtSerpin1 from *Arabidopsis* and BSZx from barley, for example, are able to inhibit a wide range of serine and cysteine proteinases (Dahl et al., 1996a) and are involved in different biotic and abiotic stresses (Alvarez-Alfageme et al., 2011; Rustgi et al., 2017; Lema Asqui et al., 2018).

**Figure 10:**
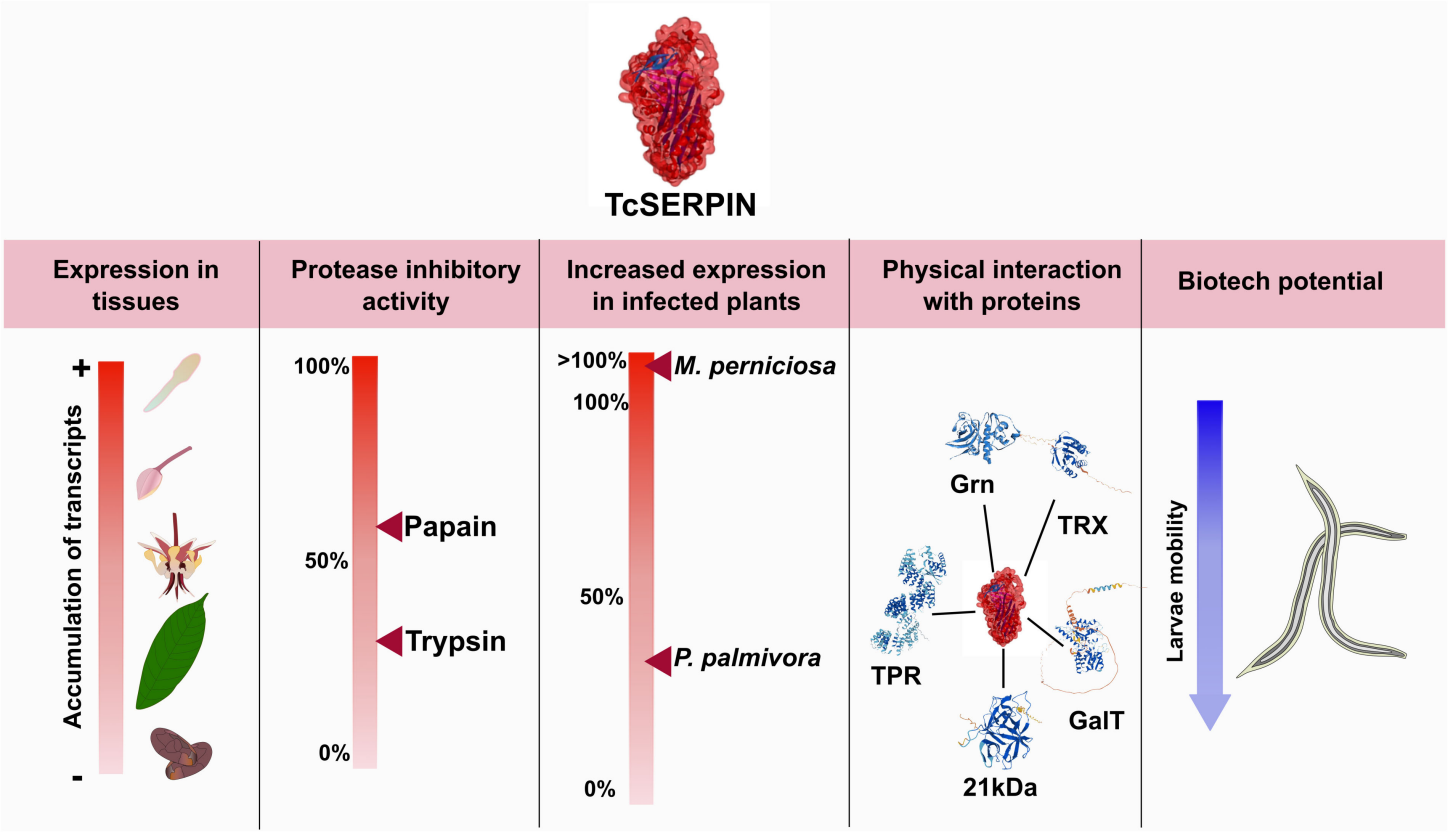
Overall scheme of the results observed for TcSERPIN. The figure shows the transcriptional profile of TcSERPIN in different cocoa tissues and in plants infected by *Moniliophthora perniciosa* and *Phytophthora palmivora*, the inhibitory activity against papain and trypsin, the capture of endogenous proteins and the evaluation of the biotechnological potential against geohelminth larvae.

In this experiment, the ‘LR’ serpin TcSERPIN, showed affinity for interaction with papain-type cysteine proteases, had its expression altered by cocoa phytopathogens and was widely expressed in different organs of healthy cocoa plants. In addition, the biotechnological potential of rTcSERPIN was evaluated, and the results bring a new perspective to this inhibitor against geohelminth larvae that cause skin infections in humans (**Figure 10**).

### 4.2 Serpins were expressed in different plant organs and are regulated by biotic stress

TPM analyses revealed that TcSERPIN transcripts were found in leaves, flowers, buds, and especially pistils (**Figure 3A**). Transcription of the *TcSERPIN* gene was conditional on stress events and possibly as a plant defense response, as observed by the increase in the TPM value caused by the fungus *M. perniciosa* when it infected *T. cacao* (**Figure 3B**), as well as by the identification of the regulatory cis-elements related to hormonal pathways, defense and stress (**Figure 1**; **Supplementary Table 2**).

The expression of serpins through stress and defense response is well known in the literature. Among the observed examples are the serpins Z4 and Z7 in barley, which were up-regulated in response to the pathogenic fungus *Fusarium culmorum* (Petti et al., 2010; Kosová et al., 2017), *MtPiI4* from *Medicago truncatula* and *AtSRP4* and *AtSRP5* from *A. thaliana* were induced by the bacterial pathogen *Pseudomonas syringae* (Sun et al., 2015; Bhattacharjee et al., 2017), and *OsSRP-ZXA* and *OsSRP-LRS* from rice were up-regulated by the fungi *Magnaporthe oryzae* and *Rhizoctonia solani*, respectively (Bhattacharjee et al., 2015; Távora et al., 2021). In wheat, several serpin genes have been affected in response to different fungal pathogens (Benbow et al., 2019). Moreover, the expression of these serpins are also conditioned to stresses caused by UV, salt (Bhattacharjee et al., 2015; Bhattacharjee et al., 2017), osmotic stress (Chen and Fluhr, 2018) and water stress (Dhanushkodi et al., 2018).

The results of this study combined with the data in the published literature (Lampl et al., 2013; Bhattacharjee et al., 2015; Bhattacharjee et al., 2017; Dhanushkodi et al., 2018) shows that the expression of *TcSERPIN* may be under regulation of different stresses and acting in the plant defense.

### 4.3 TcSERPIN was thermostable at pH 8

Circular dichroism (CD) is a sensitive technique for monitoring conformational changes in proteins and is generally used to determine the secondary structure of macromolecules (Zhang et al., 2018). In this context, the CD spectra of the rTcSERPIN protein in its native state revealed that its structure mainly had β sheet characteristics, with a negative sign at 216 and 217 nm, compatible with the expected standard spectra of this structure, according to the oligo- and polypeptide model (Lima et al., 2014).

However, possible conformational changes were observed in this protein with temperature and pH variation. At pH 7, heating the protein modified the signal and caused a shift of the negative peak from 216 nm (-33.5 mdeg) to 218 nm (-24.6 mdeg). These pH changes had significant effects on the protein structure, although rTcSERPIN still maintained β sheet formation at pH 7 (95°C). The signal increase suggests that the protein undergoes conformational changes induced by pH and temperature.

Other attempts to analyze the rTcSERPIN spectrum at pHs below 7, in citrate buffer (10 mM), did not show satisfactory results, since the protein signal was similar to the signal it exhibited at 95 °C and pH 7 (**Supplementary Figures 4B, C**). In addition, attempts to dialyze rTcSERPIN in other buffers commonly used in the laboratory, with more acidic pHs, did not return positive results and the recombinant protein precipitated considerably (data not shown). This made it impossible to obtain rTcSERPIN at a concentration of 200 µg/mL, which is the minimum concentration recommended for analysis by circular dichroism. It appears that rTcSERPIN is not stable in citrate buffer or at pH much lower than 7.

Previous observations have shown that serpins from barley and wheat grains, for example, are apparently inactivated at more acidic pH levels (Roberts et al., 2003). Changes in acidity and temperature are efficient strategies for inactivating plant defense proteins during *Theobroma cacao-Moniliophthora perniciosa* interaction (Freitas et al., 2015).

Serpins are metastable proteins. With changes in temperature and pH, serpins can change their structure by folding into a metastable state that enables the insertion of the RCL into its β sheet A, an important step for the mechanism of proteinase inhibition. However, an undesirable consequence of this action is the fact that serpins can form polymers simply by the ability to insert the loop of one serpin between the βA sheets of another. This can occur due to mutations in specific regions of the loop, but also in response to an increase in temperature. Heating between 50 – 60°C can cause this state of polymerization in serpins (Gettins, 2002). In our analyses at pH 7 heating above 66 °C caused progressive denaturation of rTcSERPIN, and consequent polymerization (**Supplementary Figure 4A**).

However, at pH 8, heating rTcSERPIN to 95 °C did not cause loss of secondary structure, showing molar ellipticity at 217 nm (-30.4 mdeg), similar to that found at 25°C (-31.2 mdeg). Resistance to temperature rise has also been observed in some barley serpins, such as Serpin-Z4 and Serpin-Z7, during preparation of beer wort (Fasoli et al., 2010; Picariello et al., 2015). Serpins found in beer show resistance to proteolysis and maintain solubility even after drastic heat treatments during the wort brewing step (Hejgaard, 1982).

### 4.4 TcSERPIN had higher affinity against cysteine proteases

*In vitro* inhibitory analysis of rTcSERPIN against serine and cysteine proteases revealed that serpin had the strongest inhibition against papain from *C. papaya*, of 54.6%, at a molar ratio of 0.65 (**Figure 5A**). As observed in the capture analyses, rTcSERPIN apparently had greater affinity for proteases that have a cysteine in their active site.

The evaluation of the capture of rTcSERPIN immobilized on CNBr-Sepharose resin with total leaf extract revealed five candidate cocoa tree proteins for an *in vivo* interaction with the serpin: 21 kDa seed; Granulin repeat cysteine protease family protein; thioredoxin-dependent peroxiredoxin; galactosyltransferase 13; and Tetratricopeptide repeat (TPR)-like superfamily protein.

The identification of the 21 kDa inhibitor may reveal a new mechanism of interaction between TcSERPIN and the cocoa Kunitz-type inhibitor that is not yet known. However, the noteworthy abundance of this protein in cocoa tissues may explain its presence by unspecific interaction. After all, the 21 kDa seed protein is among the most abundant protein in the total leaf extract, compared to cysteine protease and thioredoxin-dependent peroxiredoxin, which were much less abundant, and galactosyltransferase 13 and tetratricopeptide repeat protein (TPR), whose abundance values were so low that they could not be identified in the total leaf samples by LC-MS/MS (**Supplementary Table 4**).

Galactosyltransferase and tetratricopeptide repeat protein (TPR) act in the energy metabolism and hormonal responses of plants, respectively. The observed interaction of rTcSERPIN with these proteins may signify a yet unknown non-inhibitory mechanism for cacao serpin. This is the case of barley, for example, where a non-inhibitory interaction of Z4 serpin with β-amylase favored an increase in the activity of the enzyme, which was previously unknown (Cohen and Fluhr, 2018).

Despite the different proteins identified, thioredoxin-dependent peroxidoxin and cysteine protease with granulin and papain domain are enzymes that have a cysteine residue in their active site. The first protein is a peroxidase related to antioxidant defense, since it reduces hydrogen peroxide (H_2_O_2_) using a peroxidatic cysteine, protecting cells against the toxicity of reactive oxygen species (ROS) and reactive nitrogen species (RNS) (Knoops et al., 2016; Zhang et al., 2020). The second protein is a protease containing granulin domain and has been implicated in plant cell death mechanisms under stress (Shindo et al., 2012; Lampl et al., 2013; Rustgi et al., 2017; Lema Asqui et al., 2018).

Although thioredoxin-dependent peroxidoxin is not a protein that has been related to plant serpins, little is known about the targets of serpins in plants. To date, endogenous targets that have been documented are RD21 and metacaspases 1 and 9 for AtSerpin1 from *A. thaliana* (Vercammen et al., 2006; Lampl et al., 2013; Rustgi et al., 2017; Lema Asqui et al., 2018; van Midden et al., 2021; Ferreira et al., 2023), and the enzyme β-amylase with serpin Z4 from barley (Cohen and Fluhr, 2018). Therefore, it is likely that the thioredoxin-dependent peroxidoxin and the other proteins identified according to coupling and mass spectrometry can be candidates for an *in vivo* interaction with TcSERPIN from cocoa that have not yet been documented.

However, among the captured proteins, the granulin-domain cysteine protease was the only identified protease. This proteinase is homologous to RD21, with 73% identity (**Supplementary Figure 5**). In *Arabidopsis*, the interaction between AtSerpin1 and RD21 exert influence on plant cell death upon biotic and abiotic stresses (Lampl et al., 2013; Koh et al., 2016; Cohen et al., 2019). RD21 is a protease found in endoplasmic reticulum (ER) bodies and vacuoles (Yamada et al., 2001; Lampl et al., 2013), and is positively regulated during dehydration (Koizumi et al., 1993), and in senescent leaves (Yamada et al., 2001).

The interaction between AtSerpin1 and RD21 has been well explored in the literature (Lampl et al., 2013; Koh et al., 2016; Rustgi et al., 2017). Studies with these proteins have demonstrated that fungal elicitors and the singlet oxygen photosensitizer, acridine orange can cause rupture of the vacuole membrane and lead to the formation of a complex between cytoplasmic serpin and vacuolar RD21 (Lampl et al., 2013; Koh et al., 2016). Treatments with detached leaves of *Arabidopsis* with overexpression of *AtSerpin1* and knockout of the *RD21* gene showed an increase in leaf decomposition after inoculation with the necrotrophic fungi *Botrytis cinerea* and *Sclerotina sclerotiorum* (Lampl et al., 2013). Similarly, overexpression of AtSerpin1 in leaves subjected to photodynamic treatment with acridine orange or drought induction repressed cell death, whereas knockout plants of RD21 were more susceptible (Koh et al., 2016).

Apparently, the interaction of rTcSERPIN with the probable cocoa papain is not random. Since this interaction occurred *in vivo*, the docking analysis offered clues that the P_2_ site (Leu_349_) may be the main site of interaction for the formation of the serpin-papain complex (**Figure 8D**). In addition, the network analyses carried out according to homology with *A. thaliana* corroborated the cited reports, in which there was close interaction of AtSerpin1 with RD21, as well as with other proteins involved in metabolic, regulatory and plant stress processes (**Figure 7**).

The interaction of AtSerpin1 with the network proteins performs different regulatory functions, ranging from stress response to regulation of phosphorylation, protein folding and catabolic process of proteasomal proteins. According to the PPI network, the orthologous protein of TcSERPIN, AtSerpin1, is also related to other cysteine proteases of the metacaspase family (MC4 and MC9). In a study carried out by (Lema Asqui et al., 2018), metacaspase 1 (AtMC1) and AtSerpin1 were located in the cytoplasm and complexes between inhibitor-protease were observed. Furthermore, double knockout of AtMC1 and AtSerpin1 increased cell death, while serpin overexpression reduced cell death in plants challenged with the bacterial pathogen *Pseudomonas syringae* (Lema Asqui et al., 2018).

In addition to the observed interactions with cysteine-like proteases, the PPI network also showed that AtSerpin1 has interactions with a subtilisin-like serine protease, S1P (SBT6.1), which is associated with salt stress regulation (Liu et al., 2007), and RGF6, a GOLVEN class protein (GLV) which is related to the regulation of root development and gravitropism (Meng et al., 2012; Whitford et al., 2012), and modulation of auxin distribution (Whitford et al., 2012).

Taken together, the results raise the hypothesis that TcSERPIN may inhibit the functions of different proteases, and that it is probably involved in the mechanisms of cell death caused by stress, in which its expression can control proteases involved in this mechanism.

### 4.5 rTcSERPIN had biotechnological potential against geohelminth larvae

The analysis of rTcSERPIN against *M. perniciosa* did not show inhibition of mycelial growth of the fungus (**Supplementary Figure 7**), unlike what was observed for TcCYS4 (Pirovani et al., 2010). In *Arabidopsis*, overexpression of AtSerpin1 and knockout of papain RD21 increased cell death caused by the hemibiotrophic fungal pathogen *Colletotrichum higgisianum*, an effect opposite to that observed against the necrotrophic fungi *B. cinerea* and *S. sclerotiorum*, in which overexpression of AtSerpin1 and knockout of RD21 reduced cell death (Lampl et al., 2013). These results may indicate that ‘LR’ type serpins such as TcSERPIN and AtSerpin1 have no effect on hemibiotrophic fungi such as *M. perniciosa* and *C. higgisianum*. It is likely that the infection mechanism of these fungi explain why these proteins have no effect on the development of these pathogens.

In this work, we observed a new perspective for the TcSERPIN and TcCYS4 inhibitors. Against geohelminth larvae, the recombinant proteins were able to interfere with their motility (**Figure 9**). This is an interesting finding, since inhibitors of the serpin or cystatin type have been widely studied against fungi and insects that are potential pathogens in the plant kingdom (Pirovani et al., 2010; Alvarez-Alfageme et al., 2011; Lampl et al., 2013; Johnson et al., 2016).

In plants, the biotechnological potential of cystatins and serpins has long been known. Cystatins, for example, are potential proteins to control pests and pathogens, with the ability to inhibit fungal mycelial growth and reduce the activity of cysteine proteases in insect extracts (Pirovani et al., 2010; Martinez et al., 2016; Premachandran and Srinivasan, 2023). Serpins have biotechnological potential not only against fungi and bacteria (Lampl et al., 2013; Sun et al., 2015; Lema Asqui et al., 2018), but also against insect larvae such as *Spodoptera littoralis*, *Helicoverpa zea* and *Acyrthosiphon pisum* nymphs (Alvarez-Alfageme et al., 2011; Johnson et al., 2016).

Geohelminths are nematodes present in the soil that affect humans by penetration of their larvae through the skin or oral ingestion of their eggs, causing disease in affected individuals (Prieto-Pérez et al., 2016). The inhibitory potential exhibited by cocoa inhibitors to geohelminth larvae is an indication that their biotechnological potential can also be explored for parasites that cause dermal lesions in humans, such as cutaneous larva migrans.

## 5 Conclusion

The present study confirms that TcSERPIN is a serpin-like inhibitor of *T. cacao* and is widely located in different parts of the plant, with TcSERPIN transcripts being more expressed under biotic stress caused by *M. perniciosa*. The synthetic model rTcSERPIN has greater thermal stability at pH 8, and a higher inhibitory percentage against papaya papain *in vitro*. Furthermore, using rTcSERPIN as bait, we identified some defense proteins present in cocoa leaves that have a cysteine residue in their active site, such as thioredoxin-dependent peroxidoxin, an antioxidant enzyme, and the cysteine protease with granulin domain, homologous with *Arabidposis* RD21, which has been identified as a protease involved in cell death under different biotic and abiotic stresses. According to these results, these proteins are strong candidates for interacting with TcSERPIN.

We also observed that rTcSERPIN affects the movement of geohelminth larvae, which cause cutaneous larva migrans, showing that this protein also has biotechnological potential against these parasites. The set of results observed in this study highlights that TcSERPIN, the first serpin to be characterized in cocoa, is a protein with potential to be explored in studies with biotechnological applications against phytopathogens and/or diseases that affect humans.

## Data availability statement

The original contributions presented in the study are publicly available and included in the article/**Supplementary Material**. This data can be found here: https://massive.ucsd.edu/ProteoSAFe/dataset.jsp?accession=MSV000093675. Further inquiries can be directed to the corresponding author..

## Author contributions

MF: Conceptualization, Data curation, Formal Analysis, Investigation, Methodology, Validation, Visualization, Writing – original draft. KF: Investigation, Methodology, Writing – review & editing. MZ: Investigation, Writing – review & editing. AA: Investigation, Methodology, Writing – review & editing. GA: Investigation, Methodology, Writing – review & editing. MS: Investigation, Writing – review & editing. AF: Methodology, Writing – review & editing. BS: Investigation, Writing – review & editing. Sd: Investigation, Writing – review & editing. IM: Conceptualization, Methodology, Writing – review & editing. AS: Conceptualization, Formal Analysis, Writing – review & editing. Md: Investigation, Methodology, Resources, Supervision, Writing – review & editing. BA: Methodology, Resources, Software, Supervision, Writing – review & editing. CP: Conceptualization, Funding acquisition, Methodology, Project administration, Resources, Supervision, Validation, Visualization, Writing – review & editing.
