# Supplementary material for: TcSERPIN, an inhibitor that interacts with cocoa defense proteins and has biotechnological potential against human pathogens
Source: Front Plant Sci. 2024 Jan 29;15:1337750. doi: 10.3389/fpls.2024.1337750 (PMC10859438; doi:10.3389/fpls.2024.1337750)
Supplement: Supplementary file 1 [file DataSheet_1.zip › Supplementary Figure 6.pdf]

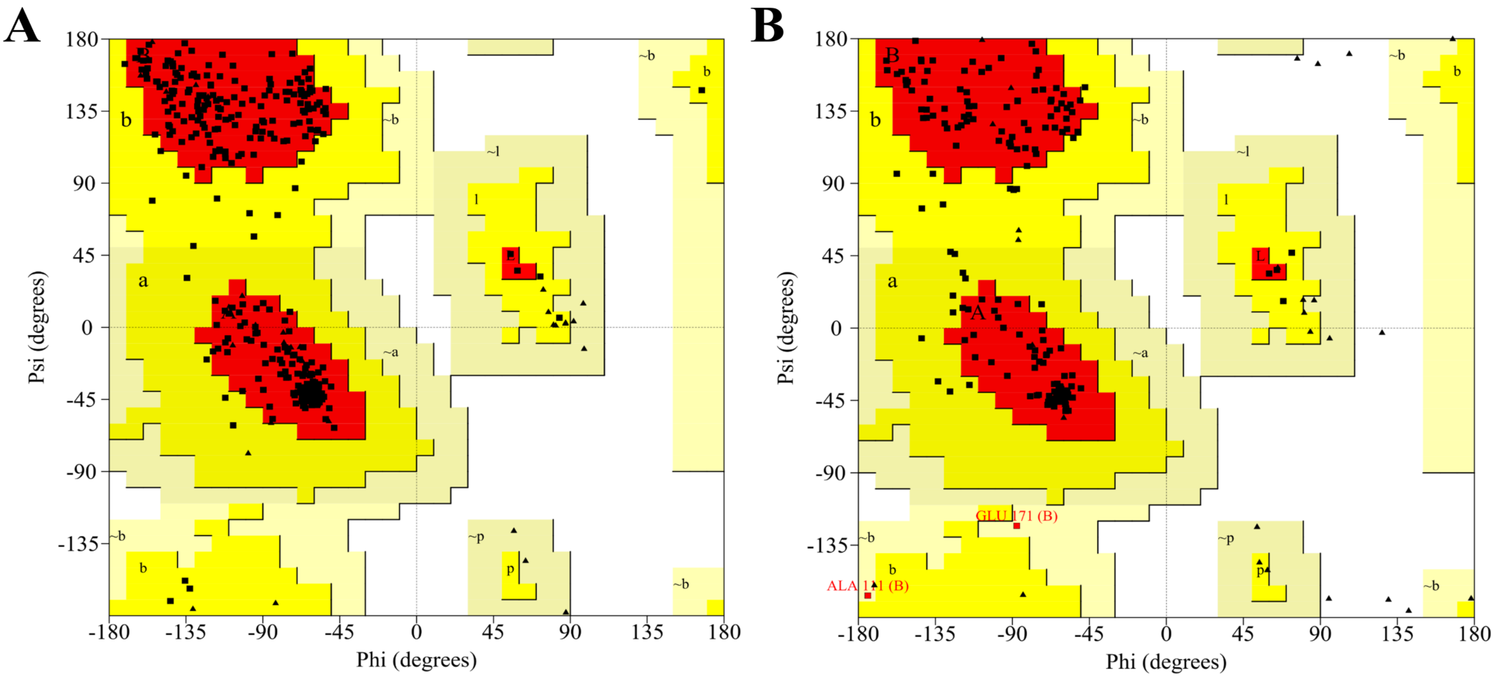

	TcSERPIN		A0A061FBU0	
**Residues in most favoured regions [A, B, L]**	314	93.7%	158	86.8%
**Residues in additional allowed regions [a, b, l, p]**	21	6.3%	22	12.1%
**Residues in generously allowed regions [~a, ~b, ~l, ~p]**	0	0.0%	2	1.1%
**Residues in disallowed regions**	0	0.0%	0	0.0%

Supplementary Material 6. Validation of the three-dimensional (3D) model built for cocoa proteins. **A** and **B**, Ramachandran plots of the 3D models generated for TcSERPIN and the cocoa cysteine protease, respectively. The regions in red, yellow, light yellow and white represent energetically more favored, permitted, generously permitted, and disallowed regions, respectively.
