# Supplementary material for: TcSERPIN, an inhibitor that interacts with cocoa defense proteins and has biotechnological potential against human pathogens
Source: Front Plant Sci. 2024 Jan 29;15:1337750. doi: 10.3389/fpls.2024.1337750 (PMC10859438; doi:10.3389/fpls.2024.1337750)
Supplement: Supplementary file 1 [file DataSheet_1.zip › Supplementary Figure 7.pdf]

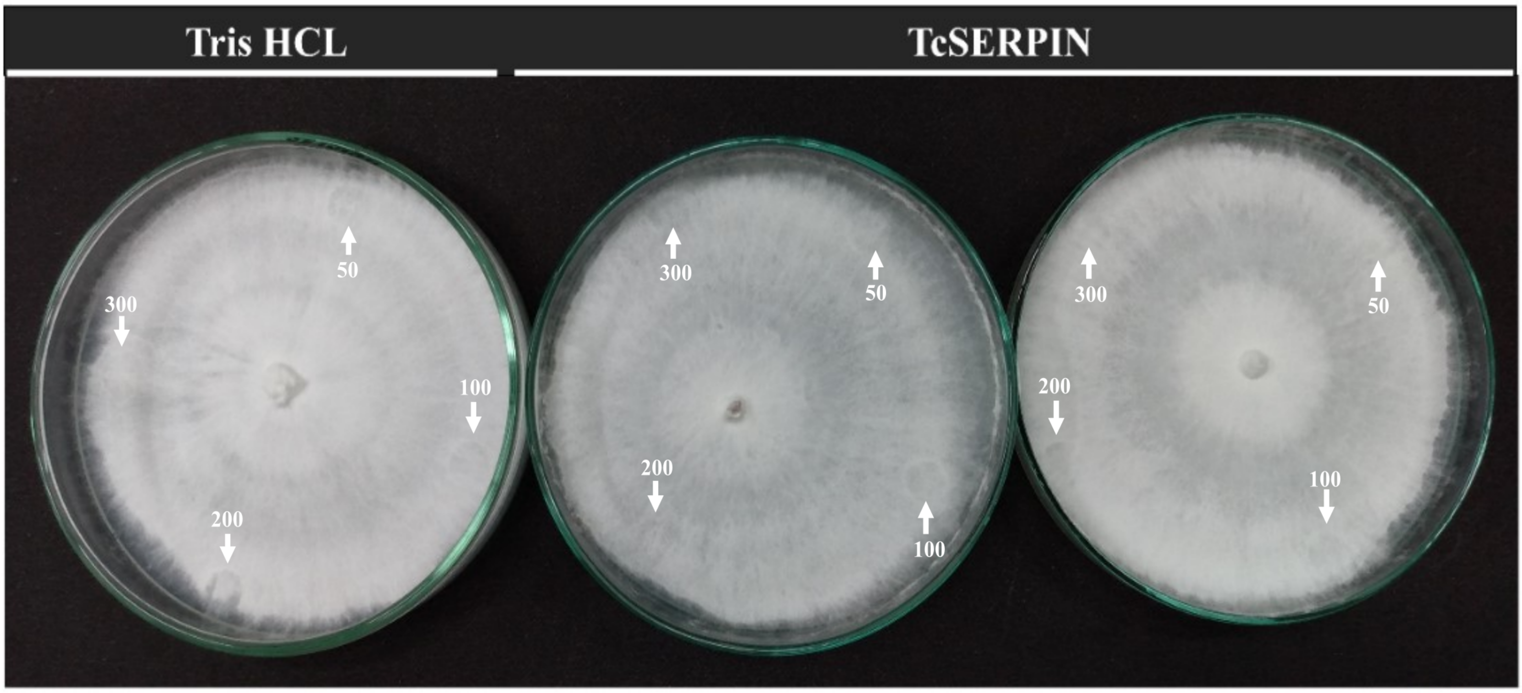

Supplementary Figure 7. Analysis of mycelial growth of *Moniliophthora perniciosa*. 22 mm mycelial discs of the *M. perniciosa* fungus isolate (CCMB 257) were placed in the center of a Petri dish containing potato dextrose agar (PDA) medium. Filter paper discs with 20 mm diameter soaked in phosphate buffered saline (PBS) containing rTcSERPIN at concentrations of 50, 100, 200 and 300 µg/mL were added to the sides of the dish for the evaluation of mycelial development under the influence of recombinant serpin. As a control, the disks were soaked in PBS without the presence of rTcSERPIN.
