# Supplementary material for: TcSERPIN, an inhibitor that interacts with cocoa defense proteins and has biotechnological potential against human pathogens
Source: Front Plant Sci. 2024 Jan 29;15:1337750. doi: 10.3389/fpls.2024.1337750 (PMC10859438; doi:10.3389/fpls.2024.1337750)
Supplement: Supplementary file 1 [file DataSheet_1.zip › Supplementary Table 1.pdf]

Supplementary Table 1. Molar ratio in each percentage of inhibition of rTcSERPIN and proteases.

rTcSERPIN X Papain (0.001067 µmols)		
**Inhibition (%)**	**rTcSERPIN in µmols**	**Molar ratio***
0	0.0000000	0.0000
14	0.0000776	0.0728
19	0.0001553	0.1455
16	0.0002329	0.2183
24	0.0003106	0.2911
22	0.0003882	0.3639
21	0.0004659	0.4366
30	0.0005435	0.5094
37	0.0006212	0.5822
55	0.0006988	0.6549
**rTcSERPIN X Trypsin (0.000840 µmols)**		
0	0.0000000	0.0000
24	0.0000518	0.0616
22	0.0001035	0.1232
21	0.0001553	0.1848
19	0.0002071	0.2464
18	0.0002588	0.3080
20	0.0003106	0.3696
20	0.0003624	0.4312
28	0.0004141	0.4928

* The molar ratio was calculated from the values in µmols of rTcSERPIN and the proteases.
