# Supplementary material for: TcSERPIN, an inhibitor that interacts with cocoa defense proteins and has biotechnological potential against human pathogens
Source: Front Plant Sci. 2024 Jan 29;15:1337750. doi: 10.3389/fpls.2024.1337750 (PMC10859438; doi:10.3389/fpls.2024.1337750)
Supplement: Supplementary file 1 [file DataSheet_1.zip › Supplementary Table 2.pdf]

Supplementary Table 2. Cis-elements present in the promoter region of *TcSERPIN* gene.

Name of Cis-elements	Organism	Sequence/Strand/Position	Function
AT~TATA-box	*Arabidopsis thaliana*	TATATA (+42, +44, +46)	Core promoter / enhancer element
CAAT-box	*Arabidopsis thaliana*	CCAAT (+257, -1300, -1443)	Common cis-acting element in promoter and enhancer regions
	Nicotiana glutinosa	CAAT (-93, -149, -265, +258, +400, -487, +613, -742, +1099)	
	Pisum sativum	CAAAT (-58, -412, +463, +601, +731, -1066, +1149, +1410)	
TATA	*Arabidopsis thaliana*	TATAAAAT (-181)	Core promoter / enhancer element
TATA-box	*Arabidopsis thaliana*	taTATAAAtc (-40)	Transcription start
	Arabidopsis thaliana	TATA (+48, +185, +193, +243, +346, +458, -975, - 354, -1414)	
	Brassica oleracea	ATATAA (+242, +1413)	
	Helianthus annuus	TATAAA (-183, -191, -456)	
	Pisum sativum	TATAAAA (-182)	
	Brassica juncea	TATAAAT (-190)	
	Brassica napus	ATATAT (+41, +43, +45, +47)	
	Arabidopsis thaliana	TATAA (-184, -192, -457)	
	Avena sativa	TATATTTATATTT (+42)	
	Brassica napus	TATATA (+44, +46)	
	Arabidopsis thaliana	TATTTAAA (+105, -107)	
ARE	*Zea mays*	AAACCA (+998)	Cis-acting regulatory element essential for the anaerobic induction

Box 4	*Petroselinum crispum*	ATTAAT (+85, +89, +114)	Part of a conserved DNA module involved in light responsiveness
ERE	*Nicotiana glutinosa*	ATTTTAAA (-126)	Ethylene-responsive element
G-box	*Petroselinum crispum*	ATTAAT (+608)	Part of a conserved DNA module involved in light responsiveness
GATA-motif	*Arabidopsis thaliana*	AAGATAAGATT (+1364)	Part of a light responsive element
GT1-motif	*Avena sativa*	GGTTAAT (-653), GGTTAA (-654)	Light responsive element
MSA-like	*Catharanthus roseus*	TCAAACGGT (+1164)	Cis-acting element involved in cell cycle regulation
MYC	*Arabidopsis thaliana*	CATTTG (+361, +411, -493, -601, +1065)	Cis-acting regulatory element involved in early response to drought and abscisic acid induction
STRE	*Arabidopsis thaliana*	AGGGG (+482)	Stress response element
Sp1	*Oryza sativa*	GGGCGG (-1224)	Light responsive elemento
TC-rich repeats	*Nicotiana tabacum*	GTTTTCTTAC (-319)	Defense and stress responsiveness
TCT-motif	*Arabidopsis thaliana*	TCTTAC (-1070)	Light responsive elemento
W box	*Arabidopsis thaliana*	TTGACC (-1162)	Sequence recognized by WRKY DNA binding proteins
WRE3	*Pisum sativum*	CCACCT (+965)	Putative element of wound response
