# Supplementary material for: TcSERPIN, an inhibitor that interacts with cocoa defense proteins and has biotechnological potential against human pathogens
Source: Front Plant Sci. 2024 Jan 29;15:1337750. doi: 10.3389/fpls.2024.1337750 (PMC10859438; doi:10.3389/fpls.2024.1337750)
Supplement: Supplementary file 1 [file DataSheet_1.zip › Supplementary Table 4.pdf]

Supplementary Table 4. Abundance of Scavina 6 genotype leaf proteins identified by LC-MS/MS.

Protein	[SC]*	Protein Name
A0A061F263	127396766.00	Thylakoid lumen 18.3 kDa protein
A0A061ECJ8	98903164.00	Glyceraldehyde-3-phosphate dehydrogenase
A0A061G8R2	98708145.00	Polyketide cyclase/dehydrase and lipid transport superfamily protein isoform 1
E3VU39	97769375.00	Photosystem I iron-sulfur center
A0A061G7Z6	96008516.00	Phosphoribulokinase
A0A061FNK9	91430305.00	2-oxoglutarate dehydrogenase, E1 component
A0A061GHX3	86068516.00	Aminomethyltransferase
A0A061F4T4	71045526.00	Fructose-bisphosphate aldolase
A0A061GRX0	65929414.00	TCP-1/cpn60 chaperonin family protein
A0A061EHN3	65242074.00	Chloroplast splicing factor CRS1, putative isoform 1
A0A061GX48	62704254.00	RuBisCO large subunit-binding protein subunit alpha isoform 1
A0A061G750	61159605.00	NAD(P)-binding Rossmann-fold superfamily protein isoform 1
A0A061F1U6	57872793.00	Superoxide dismutase [Cu-Zn]
A0A061FL99	55506066.00	Catalase
A0A061GY96	55024673.00	Lactoylglutathione lyase
A0A061FZH7	53166557.00	Cytochrome P450, family 71, subfamily A, polypeptide 25, putative
A0A061F3W4	45253876.00	Chloroplast RNA binding
A0A061G4W3	44665665.00	Expansin-like B1, BETA 3.1,EXLB1
A0A061FN17	43773076.00	Uncharacterized protein isoform 2
A0A061FK63	43138737.00	Ferritin
A0A061ECL0	42141605.00	Serine/threonine-protein kinase STN7
A0A061GDG9	42093136.00	NAD(P)-binding Rossmann-fold superfamily protein isoform 3
A0A061FC81	38745876.00	Cytochrome P450 82A3, putative
A0A061GI46	38139835.00	DDB1-CUL4 associated factor 1
A0A061DWB5	37957237.00	Malate dehydrogenase
A0A061ECX5	35954166.00	Isocitrate dehydrogenase [NADP]
A0A061ETB9	34496619.00	Ser/arg-rich protein kinase 4 isoform 1
A0A061FPM8	34492256.00	CRINKLY4 related 2
A0A061DU83	33983555.00	Rubisco methyltransferase family protein isoform 1
A0A061GU06	32586826.00	Xanthine dehydrogenase 1 isoform 1
A0A061GUD0	32442413.00	Lactoylglutathione lyase
A0A061EAS0	32284252.00	NADPH-protochlorophyllide oxidoreductase
A0A061DX77	32241394.00	Malate dehydrogenase
A0A061EGH6	31653822.00	zf-RVT domain-containing protein
A0A061GL19	31556381.00	Chaperonin 20 isoform 1
A0A061GG81	30535923.00	Acyl-CoA sterol acyl transferase 1, putative
A0A061DVC2	29873917.00	MIF4G domain-containing protein / MA3 domain-containing protein isoform 1
A0A061FTB4	28654232.00	Plasma membrane intrinsic protein 1,4
A0A061GVV2	21083013.00	Aluminum activated malate transporter family protein, putative
A0A061F5V2	18115854.00	rRNA N-glycosidase
A0A061G4Z2	16437036.00	Protein kinase domain-containing protein
A0A061GL56	15937812.00	Cysteine proteinases superfamily protein

A0A061FCH5	15085394.00	Uncharacterized protein
A0A061DNS9	14170178.00	PSI subunit V
A0A061E7Y6	13228365.00	Protein SDA1
A0A061DMJ4	13207788.00	Chlorophyll a-b binding protein, chloroplastic
A0A061E0H3	11971978.00	Thylakoid rhodanese-like, putative isoform 1
A0A061FUG0	10482505.00	Aldehyde dehydrogenase family 2 member B4
A0A061EC97	8631501.00	Uncharacterized protein
A0A061FA92	8006951.00	5'-3' exoribonuclease 3
A0A061DM75	6720904.00	RING-type E3 ubiquitin transferase
A0A061FAA5	6299243.00	RNA helicase
A0A061DWF1	6253762.00	Mog1/PsbP/DUF1795-like photosystem II reaction center PsbP family protein
A0A061FEJ5	5888504.00	Transducin/WD40 repeat-like superfamily protein isoform 4 (Fragment)
A0A061EXZ0	5514398.00	Uncharacterized protein
A0A061DSR0	5432903.00	Alpha/beta-Hydrolases superfamily protein
A0A061G4F3	4209465.00	Pentose-5-phosphate 3-epimerase
A0A061G4X7	4207433.00	Uncharacterized protein
A0A061E2R1	4202096.00	Pectin lyase-like superfamily protein isoform 1
A0A061G9X7	4169259.00	Uncharacterized protein
A0A061GXA3	3945404.00	RNA helicase, ATP-dependent, SK12/DOB1 protein isoform 1
A0A061EX41	3768389.00	Dihydrolipoyl dehydrogenase
A0A061FYL6	3307848.00	Haloacid dehalogenase-like hydrolase superfamily protein
A0A061GG51	3264105.00	Photosystem II subunit Q-2
A0A061GTT9	2752584.00	Alanine--glyoxylate aminotransferase
A0A061FUY8	2065966.00	Elongation factor Tu
A0A061EDR8	1272944.00	Protein FAR1-RELATED SEQUENCE
A0A061FWL5	862023.20	21 kDa seed protein
A0A061GAL9	717433.75	Duplicated homeodomain-like superfamily protein, putative
A0A061FDN2	572822.80	Plastocyanin
E3VTZ5	570347.20	Ribulose bisphosphate carboxylase large chain
A0A061EH79	527065.10	Ribulose bisphosphate carboxylase small chain
A0A061DSX4	483648.16	Photosystem II subunit P-1
A0A061F7Z6	361506.00	PR5-like receptor kinase
A0A061DG44	340045.90	Photosystem II subunit O-2
A0A061E001	338142.40	Glycerate dehydrogenase isoform 1
E3VU04	320814.94	Cytochrome b559 subunit alpha
A0A061FI76	299121.97	Alcohol dehydrogenase 1 isoform 1
E3VTW8	292192.75	Photosystem II protein D1
A0A061ERE1	268768.94	Histone H2A
A0A061GPF6	263088.72	Histone superfamily protein
E3VU13	249690.10	Photosystem II CP47 reaction center protein
A0A061GD38	243506.77	Chlorophyll a-b binding protein, chloroplastic
A0A061GXD0	232809.55	Clathrin heavy chain
E3W0D7	228352.55	Photosystem II reaction center protein H
A0A061DJA0	225484.84	Actin 7 isoform 1
A0A061EKX7	216514.75	Photosystem I subunit D-2
A0A061ETP3	214564.22	GTP binding Elongation factor Tu family protein

A0A061EM25	213073.92	Plastid-lipid associated protein PAP / fibrillin family protein
A0A061EVI6	199338.95	Thioredoxin-dependent peroxiredoxin
E3VTY4	182144.73	Photosystem II CP43 reaction center protein
A0A061EQW6	175395.69	Chlorophyll a-b binding protein, chloroplastic
A0A061DYK6	170066.56	Fructose-bisphosphate aldolase
A0A061FPW8	168212.60	Thioredoxin M-type 4
E3VTX3	164743.56	ATP synthase subunit alpha
A0A061FMD6	161859.27	Rubisco activase isoform 2
E3VTZ4	160995.14	ATP synthase subunit beta
A0A061GD16	160704.02	PSI-F
A0A061FHZ0	160194.90	Chlorophyll a-b binding protein, chloroplastic
A0A061GG32	154810.39	Plastid-lipid associated protein PAP / fibrillin family protein
A0A061FB18	151118.06	Phosphoglycerate kinase
A0A061GGN9	149857.88	PPO1_KFDV domain-containing protein
E3VTY3	149690.95	Photosystem II D2 protein
A0A061FA06	148764.67	(S)-2-hydroxy-acid oxidase
A0A061F1M8	140384.47	Photosystem I subunit E-2-like protein
A0A061DIX8	140225.50	Serine hydroxymethyltransferase
E3VTY7	133228.08	Photosystem I P700 chlorophyll a apoprotein A1
E3VU00	132090.19	Cytochrome f
A0A061ETY3	131838.60	Glutaredoxin-dependent peroxiredoxin
A0A061F5T3	128756.62	rRNA N-glycosidase
A0A061EDL4	127778.99	Glyceraldehyde-3-phosphate dehydrogenase
A0A061E0B2	125070.38	Peptidyl-prolyl cis-trans isomerase
A0A061FJ08	122892.37	Arginase isoform 1
A0A061GWC1	122660.74	Superoxide dismutase [Cu-Zn]
A0A061FLC9	109792.87	Sedoheptulose-bisphosphatase
A0A061ECM1	103851.85	Carbonic anhydrase
A0A061GXG8	103815.06	Histone H2B
A0A061F2B8	102816.18	Cysteine synthase
A0A061F909	102511.47	Photosystem II 10 kDa polypeptide, chloroplastic
A0A061F976	100784.38	Pathogenesis-related protein P2
A0A061FBU0	100244.99	Granulin repeat cysteine protease family protein
A0A061E3S5	98772.49	Plastoquinol--plastocyanin reductase
A0A061GVQ7	94867.14	Plastid transcriptionally active 16
A0A061ERA1	93250.59	Histone H4 (Fragment)
A0A061EM86	91322.43	Glutamine synthetase
A0A061GSW1	86385.89	Lipoxygenase
E3VTY6	85181.47	Photosystem I P700 chlorophyll a apoprotein A2
A0A061DV41	79464.81	Transketolase
A0A061GRY8	77938.87	Glycine cleavage system P protein
A0A061DFA2	74042.87	Ferredoxin--NADP reductase, chloroplastic
A0A061GFT3	72790.59	ATPase, F1 complex, gamma subunit protein
A0A061E9U2	68280.73	Nodulin-related protein 1, putative
A0A061FN40	67340.84	PsbP-like protein 1 isoform 1
A0A061FHB6	65945.10	Chlorophyll A-B binding family protein

A0A061F6X3	60859.55	Chloroplastic lipocalin
A0A061E033	1828.83	Granulin repeat cysteine protease family protein
A0A061FVK5	1.00	21 kDa seed protein
A0A061F828	1.00	NADPH-protochlorophyllide oxidoreductase
A0A061FDM1	1.00	Isoflavone reductase-like protein 4 isoform 1
A0A061EZI5	1.00	5-methyltetrahydropteroyltriglutamate--homocysteine S-methyltransferase
A0A061EPV7	1.00	S-adenosylmethionine synthase
A0A061GEN3	1.00	Larreatricin hydroxylase
A0A061E5Y3	1.00	Major latex protein, putative
A0A061DXT6	1.00	Tudor/PWWP/MBT domain-containing protein, putative isoform 2
A0A061DQG4	1.00	Multidrug resistance-associated protein 4 isoform 1
A0A061EJ28	1.00	Chloroplast heat shock protein 70 isoform 3
A0A061DRW3	1.00	Reduced epidermal fluorescence 4, putative isoform 1
A0A061E5F2	1.00	DUF4220 domain-containing protein
A0A061F0S1	1.00	Pentatricopeptide repeat-containing protein, putative isoform 3
A0A061GES8	1.00	Mg-protoporphyrin IX chelatase
A0A061EZQ5	1.00	Uncharacterized protein isoform 1
A0A061GK06	1.00	Sucrose synthase
A0A061E4T6	1.00	RNase H domain-containing protein
A0A061ED44	1.00	Auxin signaling F-box 2
A0A061EPA5	1.00	Ribosomal protein L14p/L23e family protein
A0A061FPU9	1.00	Phosphoenolpyruvate carboxylase family protein
A0A061FTN7	1.00	LOB domain-containing protein 29
A0A061EQW5	1.00	Tautomerase/MIF superfamily protein
A0A061GCR4	1.00	B-block binding subunit of TFIIIC, putative
A0A061GNU8	1.00	Non-specific serine/threonine protein kinase
A0A061G5J8	1.00	Ribosomal protein S13A, putative
A0A061G2D1	1.00	Caffeic acid 3-O-methyltransferase 1
A0A061F630	1.00	ATPase, F1 complex, gamma subunit protein
A0A061GDG7	1.00	Eukaryotic translation initiation factor 4A1 isoform 1
A0A061E4I1	1.00	Ribosomal protein L22p/L17e family protein isoform 1
A0A061ER16	1.00	NAD(P)-binding Rossmann-fold superfamily protein isoform 1
A0A061F8V4	1.00	Ribosomal protein S5/Elongation factor G/III/V family protein
A0A061GBW3	1.00	40S ribosomal protein SA
A0A061DSY4	1.00	Receptor-like protein kinase 4 isoform 1
A0A061E3E6	1.00	Dimer_Tnp_hAT domain-containing protein
A0A061DQS0	1.00	Photosystem I subunit D-2
A0A061ENW0	1.00	Translationally-controlled tumor protein
A0A061EU04	1.00	Fructose-bisphosphate aldolase

*The abundance was calculated by the log2 of the median intensity of the identified peptides of each protein.
