# Supplementary material for: TcSERPIN, an inhibitor that interacts with cocoa defense proteins and has biotechnological potential against human pathogens
Source: Front Plant Sci. 2024 Jan 29;15:1337750. doi: 10.3389/fpls.2024.1337750 (PMC10859438; doi:10.3389/fpls.2024.1337750)
Supplement: Supplementary file 1 [file DataSheet_1.zip › Supplementary Figure 1.pdf]

Alinhamento *TcSERPIN* X Pseudogene 3’UTR

```
Pseudogene     ------------------------------------------------------------
TcSERPIN       TCAAGTGTTCAACACGGATTCTCTCCTACGCGGCAAAGCCGCGACGGTCCCATTTCACAA

Pseudogene     ------------------------------------------------------------
TcSERPIN       TATTAAATATTCAGCTGTCCTGCTTAAAGATAACGGTCCTTGTTTCGGCTCGGCTTTGTC

Pseudogene     ------------------------------------------------------------
TcSERPIN       GAAGACTTGAGCTAAAACCTAAGTGAAGTAAAAGGGCTAACTTTGTTCTCGACTTGTTTG

Pseudogene     ------------------------------------------------------------
TcSERPIN       TGTTTTAGATTCCGACAATGGATCTCCGTGAATCGATCAGGAGGCAAACCGACGTAACCT

Pseudogene     ------------------------------------------------------------
TcSERPIN       TAAGCCTGACGAAGCACGTGCTCCAGACCGAAGCCAAGGACTCCAACCTTGCCTTCTCGC

Pseudogene     ------------------------------------------------------------
TcSERPIN       CGCTGTCGATCCACGTGGTGCTCAGCATGATCGCGGCTGGCTCTACGGGCCCAACCCTCG

Pseudogene     ------------------------------------------------------------
TcSERPIN       ACCAGCTCCTCTCTTTCCTCAAGTCAGCATCCAACGACCACCTCGGCTCCTTCTCCTCCG

Pseudogene     ------------------------------------------------------------
TcSERPIN       AGCTCGTTTCCGTTGTGTTCGCCGACGGTAGCCCCGCGGGTGGGCCCCGGTTGTCGTTTG

Pseudogene     ------------------------------------------------------------
TcSERPIN       CCAACGGGGTTTGGATCGACAAGTCTCTCCCTCTCAAGCCTTCTTTCAAACAGGTTGTGG

Pseudogene     ------------------------------------------------------------
TcSERPIN       ATAATGTCTACAAGGCTGCTTCTAATCAAGTCGATTTCCAAACCAAGGTAGTAGCTTCTT

Pseudogene     ------------------------------------------------------------
TcSERPIN       TATTTGGATTCTGATGTTTGTCTTCCGATCGCTGCTTTTAATATGTCTCTGTTCTTTCCC

Pseudogene     ------------------------------------------------------------
TcSERPIN       TTCAAATGTAGCTGGGATCTTAACACGTTGCAAAACTGGATATTTTTCAAGTTTCAAATC

Pseudogene     ------------------------------------------------------------
TcSERPIN       TATGGGGATCCAGTGACCCCAATTGTTTCCCAAATCTTATAGATGTAGCCATGCCCATAT

Pseudogene     ------------------------------------------------------------
TcSERPIN       TTTGAAAATAAGAATTTCAACACAGTACTTATGAGAAATAGTACCTTTTTTTTTTTGAAT

Pseudogene     ------------------------------------------------------------
TcSERPIN       TTTTTTTATAGATAGTACTTATCAAGCATTCCTTGAAACTGTAGATAATTAAGTATAAGC

Pseudogene     ------------------------------------------------------------
TcSERPIN       TACTTATAGGAAGGAATATATGAACTAATTCCTGTACCACTGATGTATAGGTAGTACATA

Pseudogene     ------------------------------------------------------------
TcSERPIN       AGTTAATGATATGTTTATTGATTATATTGGTAGCATTCACTTGCCATTTGTTTTTTAACT

Pseudogene     ------------------------------------------------------------
TcSERPIN       TCTGGGTTAAATGAATATGTTAACTGTTCTTCTTCTTTTTTTTAATGTTGTTGGTATCTT

Pseudogene     ------------ATGAGGCA---------------TGGGCA---------TACAGTCCAC
TcSERPIN       TGAAGGCTGTTCAAGTGGCTGGTGAAGTGAATTTGTGGGCAGAAAAGGAGACCAGTGGTC
```

```
                                *:*:***:               ******         :.****  :*

Pseudogene    A-----AACAA---AAGCCCCCA---TCGG----------------CCCACTCTAAGC--
TcSERPIN      TTATTAAACAACTTCTTCCGCCAGGGTCGGTTGATGGTTCAACCAGGCTTATATTTGCTA
              :     *****   .: ** ***   ****                 * :.*.*::**

Pseudogene    ------TTAACTCCAACGCGGCAAAGC-AGCAACGTCCTTCCTTCCCTCTTTAGAACAAC
TcSERPIN      ATGCACTTTACTTCAAAGGAGCTTGGAATGAAACGTTCG---ATGCGTCAA--AAACAAA
                    **:*** ***.* .**::.*. :*.***** *     :* * **::  .*****.

Pseudogene    AGTGCTTTGCTCTTCACTCTC-TGCTGGTGAACTCTAT---CGCATCT---AT----AGC
TcSERPIN      AGAAAATG--ACTTCTACCTTGTAAATGGAAGCTCTGTTAAGGCACCTTTTATGACCAGC
              **:..:*   :****:. **  *..: * .*.****.*    *** **   **    ***

Pseudogene    CTA----AGCAAGGCAATGTTCTTGCTTGGAGTTGAGATTTGCTTTTAGTTTCC-GAACA
TcSERPIN      CAAAAGAAGCAAGCCGTT----------GGT---GCGTA----TGATGGTTTCAAAGTCC
              *:*    ****** *.:*          **:  *.*::    * :*.*****. ..:*.

Pseudogene    ATGGACCTCCGTGAATCGATCAGATGGAGATAACCGTCGGTTCTCCTTGTACATCTGTCT
TcSERPIN      TAGGGCTTCCGTAT--AAACAAGGTGGAGATAAGCGTCGTTTCTCCATGTATTTCTTTCT
              ::**.* *****.:   ..* .**.********* ***** ******:**** :*** ***

Pseudogene    TCCAGAGGGAAAAGATGGCATGCCAAACTTTGGTAGAGTAAGCGAGTTCCGAATCTGGTT
TcSERPIN      TCCAGATGCAAAAGATGGTCTGCCG-GCTTTGGTAGAGAAAGTGAGTTCTGAATCTGGTT
              ****** * ********* .****. .***********:*** ****** **********

Pseudogene    TCTTAGAACGCCACCTTCCATCCATCTAGAACAGTAGAAGTTGGTGAATTCAGGATCCCA
TcSERPIN      TCTTGGAACGCCACCTGCCAT----ATGAACCAGTTAAAGTGGGTGAATTCAGGATTCCA
              ****.*********** ****     .*..*.****:.**** ************** ***

Pseudogene    AGGCTCAAGATTTCATTCTGGTTCGAAGCTTCGGAAGTTCTCAAAGGAAGCTT---CG--
TcSERPIN      AGATTCAAGATTTCATTTGGATTCGAAGCTTCTGAAGTTCTGAAGAGATTAGGACTTGTA
              **. *************  *.*********** ******** **..**: .      *

Pseudogene    --GAGGTTCTCAAAGGAAGCTTCGGAGGTCTCAAAGGAATTAATGTCTGATTCGACTGTG
TcSERPIN      TTGCCTTTCTCTGGTGA-----AGGAGGTTTGAC----AGAGATGGTGGATTCGCCTTTG
                *.  *****:.. **     .****** * *.    * :.***   ******.** **

Pseudogene    GGTCAGAAACC-------------------------------------------------
TcSERPIN      GGTCAAAGCCTGTATGTTTCAAATATATTCCATAAATCTTTCATTGAAGTTAATGAAGAA
              *****.*..*

Pseudogene    ----TACAGGCCGCTGCTGTCACAGCAGGTCTCTACAAACTCTTCTGT--TCA--T---T
TcSERPIN      GGGACAGAAGCTGCAGCTGCTTCTGCTGGTGTTATAAGACTCAGGGGTGTGCTTGTTGAG
                   * *.** **:****  :*:**:*** * ::.*.****:   **   *:  *

Pseudogene    GCAAATGACTTTGGGGCTGCTGACCATCCTTTCCTTTTCCTGATCAGAGAAGATGTGACT
TcSERPIN      GAAAAAATAGATTTTGTGGCTGACCATCCATTCCTCTTCCTGATCAGGGAAGATGTGACT
              *.***:.:. :*   *  ***********:***** ***********.************

Pseudogene    GGCGTTGTTCTGTTCATAGGGGCATGTGCTCAATCCTCTTGAAAGCTGAACCAGCCTGCG
TcSERPIN      GGAGTTGTTCTGTTCATTG-GGCACGTCCTCAATCCTCTTGAAAGTTGATAAATCT-GTT
              **.**************:* **** ** **************** ***:..* *  *

Pseudogene    ATATACC-------GGAGAATTCGTATGAAT-G--GATCTTTCCTAGCACGTGGTAGAAG
TcSERPIN      CTGATCCTTATGTAGGAAAATGTGTACGAGGAGGATAACTAGCTGAACAAATGGAAGA--
              .*.::**       ***.***  *** **.  *    *:**: *  *.**..***:***

Pseudogene    ATTGTGGTAAAATATAACCATCTGATTAGTTTGCAGTGTTTTCCAAATGCCTGAAA----
```

```
TcSERPIN        TTTGTGGTTATT-TTAAGCATCCGGTTGATGTGTTGTAGTTTGCATATGCCAGAGCCTAG
                :*******:*:: :*** **** *.**..* ** :**. *** **:*****:**..

Pseudogene      --CCC-CAATGTGGCTTAATATTCTATA----GT-------TTAAACAGGTATATAACCT
TcSERPIN        TGCCGCTTAAGTGGTTGTGTAGTCTAACCCGTGTGACTTATGCTTTCTTGTAAGTTTCCT
                  **   :*:**** * :.** ****:.    **         :::*: ***:.*::***

Pseudogene      TTTAAATAAAAAGCTGGATCTGTACT---GATCCAATGCAA--------ATA------TT
TcSERPIN        TTTCGTTT----CCTATGTCTTTGCTGTTGATCCCAGTAGGTTATAACAATAAACTACTT
                ***..:*:     **. .*** *.**   *****.*  ...        ***      **

Pseudogene      CTTTTTCCCTCTTC-TCTTCAGATTCAG-----TGTACATGTAATATAAAGAGAG-----
TcSERPIN        GTTGTTCCCATTAGAAATACATTCTCATGGCTTTTGACTTGTTGAATACTTAAAGCTTAG
                 ** *****: *:  :.*:** : ***      *  **:***:.:***.: *.**

Pseudogene      -TCAAC--AGTA-
TcSERPIN        CTCTCCTCTCTTA
                 **:.*  : *:
```

Alinhamento *TcSERPIN* X Pseudogene 5'UTR

```
Pseudogene      ------------------------------------------------------------
TcSERPIN        TCAAGTGTTCAACACGGATTCTCTCCTACGCGGCAAAGCCGCGACGGTCCCATTTCACAA

Pseudogene      ------------------------------------------------------------
TcSERPIN        TATTAAATATTCAGCTGTCCTGCTTAAAGATAACGGTCCTTGTTTCGGCTCGGCTTTGTC

Pseudogene      ------------------------------------------------------------
TcSERPIN        GAAGACTTGAGCTAAAACCTAAGTGAAGTAAAAGGGCTAACTTTGTTCTCGACTTGTTTG

Pseudogene      -----------------ATGGATGTTCGTGAAATGATCAGGAGCCAGACCGACGTCGCTC
TcSERPIN        TGTTTTAGATTCCGACAATGGATCTCCGTGAATCGATCAGGAGGCAAACCGACGTAACCT
                                 ****** * ******: ********* **.********..*

Pseudogene      TGAGCCTCACGAAGCACGTTCTCACAACCCAAGCCGAGGTGGACTCGAACCTGGTGTTCC
TcSERPIN        TAAGCCTGACGAAGCACGTGCTCCAGACCGAAGCCAAG---GACTCCAACCTTGCCTTCT
                *.***** *********** ***...*** *****.**   ***** ***** *  ***

Pseudogene      CGCCGCTGTCTGCACAAGTGGTGCTGAGCGCCATCGCCGTTGGCTCAAATGGTGCCAACC
TcSERPIN        CGCCGCTGTCGATCCACGTGGTGCTCAGCATGATCGCGGCTGGCTCTACGGGCCCAACCC
                ********** . .**.******** ***.  ***** * ******:*. **  *.*.**

Pseudogene      TTGGCCAGCTCCTCTGCTTCCTCAAGTCGACGTCCAACGACCACCTCAGCTCCTTCTACT
TcSERPIN        TCGACCAGCTCCTCTCTTTCCTCAAGTCAGCATCCAACGACCACCTCGGCTCCTTCTCCT
                * *.***********  ***********..*.***************.*********.**

Pseudogene      CCGAGATCATTTCCGCGGTCTTTGCAGACGGCAGCCCGGTGGGTGGGCCCCGCTTGTCTT
TcSERPIN        CCGAGCTCGTTTCCGTTGTGTTCGCCGACGGTAGCCCCGCGGGTGGGCCCCGGTTGTCGT
                *****.**.******  ** ** **.***** ***** * ************ ***** *

Pseudogene      TCTCTAATGGTGTTTGGGTCGACAAGTCTCTCCCTCTCAAGCACTCCTTTAGACAGATAA
TcSERPIN        TTGCCAACGGGGTTTGGATCGACAAGTCTCTCCCTCTCAAGCCTTCTTTCAAACAGGTTG
                *  * ** ** ******.************************. ** ** *.****.*:.

Pseudogene      TGAAGAATGTCTACAAGGCTGCTTCCAATCAAGTTGATTTCCAAACCGAGGTATTATATT
```

```
TcSERPIN        TGGATAATGTCTACAAGGCTGCTTCTAATCAAGTCGATTTCCAAACCAAGGTAGTAGCTT
                **.* ******************** ******** ***********.***** ** .**

Pseudogene      T-------GC---------------------TCTCC--------------TCTGTAAT--
TcSERPIN        CTTTATTTGGATTCTGATGTTTGTCTTCCGATCGCTGCTTTTAATATGTCTCTGTTCTTT
                        *                      ** *               *****:.*

Pseudogene      ---------TTTTGT-------------------------------------TTGTTTTA
TcSERPIN        CCCTTCAAATGTAGCTGGGATCTTAACACGTTGCAAAACTGGATATTTTTCAAGTTTCAA
                         * *:*                                     ::**** :*

Pseudogene      AACTAT-------------------------------------------------GATCT
TcSERPIN        ATCTATGGGGATCCAGTGACCCCAATTGTTTCCCAAATCTTATAGATGTAGCCATGCCCA
                *:****                                                 *. *:

Pseudogene      TATCTTGTTTAGTATATTTTCTATTCG-------------ATTTTCCCTTTTTTTTT---
TcSERPIN        TATTTGAAAATAAGAATTTCAACACAGTACTTATGAGAAATAGTACCTTTTTTTTTTTG
                *** ***:::* :* *:****:* :*.             **: *.***********

Pseudogene      ------------------------------------------------------------
TcSERPIN        AATTTTTTTTATAGATAGTACTTATCAAGCATTCCTTGAAACTGTAGATAATTAAGTATA

Pseudogene      ----TCTATC----------------------------------------TGGGAA----
TcSERPIN        AGCTACTTATAGGAAGGAATATATGAACTAATTCCTGTACCACTGATGTATAGGTAGTAC
                    :**::                                         *.**:*

Pseudogene      -------------------------------------ATTCTTATGCCAATTAACTTT--
TcSERPIN        ATAAGTTAATGATATGTTTATTGATTATATTGGTAGCATTCACTTGCCATTTGTTTTTTA
                                                     ****: :*****:**.: ***

Pseudogene      ------------------------------------TTTTGTTTGTCATGTTTTTAGTTT
TcSERPIN        ACTTCTGGGTTAAATGAATATGTTAACTGTTCTTCTTCTTTTTTTTAATGTTGTTGGTAT
                                                    * ** *** *.***** **.**:*

Pseudogene      CTTTGAAGGCTGATCTCATGAGGAGTGAAGTGAACTTATGGGCGGAAAAGGAGACGAATG
TcSERPIN        CTTTGAAGGCTGTTCAAGTGGCTGGTGAAGTGAATTTGTGGGCAGAAAAGGAGACCAGTG
                ************:**:..**.  .********** **.*****.*********** *.**

Pseudogene      GTCTTATTAAACAAGTTCTTCCTCCAGGGTCTGTGAACCGTTTGACCAGGCTCATATTTG
TcSERPIN        GTCTTATTAAACAACTTCTTCCGCCAGGGTCGGTTGATGGTTCAACCAGGCTTATATTTG
                ************** ******* ******** ** .*  *** .******** *******

Pseudogene      CTAATGCACTTTACTTCAAAGGAGTTTGGAATGAAAAATTCGATTCATTGAAAACAAAAG
TcSERPIN        CTAATGCACTTTACTTCAAAGGAGCTTGGAATGAAACGTTCGATGCGTCAAAAACAAAAG
                ************************ ***********..****** *.* .**********

Pseudogene      ACCATGACTTCTATCTTACAAATGGAAGCTCTGTTCAAGTGCCCTTTATGACCAGCAAGA
TcSERPIN        AAAATGACTTCTACCTTGTAAATGGAAGCTCTGTTAAGGCACCTTTTATGACCAGCCAAA
                *..********** ***. ****************.*.* .** ************.*.*

Pseudogene      AGAAGCAGTACATTCGCGCCTATGATGGTTTCAAAGTGCTCGGACTTCCTTATAAGCAAG
TcSERPIN        AGAAGCAAGCCGTTGGTGCGTATGATGGTTTCAAAGTCCTAGGGCTTCCGTATAAACAAG
                *******. .*.** * ** ***************** **.**.***** *****.****

Pseudogene      GTGGAGATATCCGCCGTTTCACCATGTACA----TCTTCCAGATGCAAGAGATGGGTTGA
TcSERPIN        GTGGAGATAAGCGTCGTTTCTCCATGTATTTCTTTCTTCCAGATGCAAAAGATGGTCTGC
                *********: ** ******:******* :    **************.******  **.
```

```
Pseudogene      AAGCTTTGGTAGAGAAAGTGAGTTCTGAATCCTGTTTCTTGCAACGCCACATTCCATACG
TcSERPIN        CGGCTTTGGTAGAGAAAGTGAGTTCTGAATCTGGTTTCTTGGAACGCCACCTGCCATATG
                ..*****************************  ******** ********.* ***** *

Pseudogene      AACAAGTTGCAGTAGGTGAATTCCGGATGCCCAGGTTCAAAATCTCATCTGGGTTTAAAG
TcSERPIN        AACCAGTTAAAGTGGGTGAATTCAGGATTCCAAGATTCAAGATTTCATTTGGATTCGAAG
                ***.****..***.*********.**** **.**.*****.** **** ***.** .***

Pseudogene      CTTGCGAAGTTGTCAAGGGATCAGGACTTGTATCACCTTTCTCTTCTCAATAAGCAGATT
TcSERPIN        CTTCTGAAGTTCTGAAGAGATTAGGACTTGTATTGCCTTTCTCTGGT---GAAGGAGGTT
                ***  ****** * ***.*** *********** .*********  *    *** **.**

Pseudogene      TGACATAGATGGCGGATCTGCCTGAGGGTCAGAACCCGTATGTTTCAGACATATTCCATA
TcSERPIN        TGACAGAGATGGTGGATTCGCCTTTGGGTCAAAGCCTGTATGTTTCAAATATATTCCATA
                ***** ****** ****  **** :******.*.** **********.* **********

Pseudogene      AATCTTTCATCGAGGTTAATGAAGAAGGGACAGAAGCTGCAGCTTGTACTGCTGCTATTG
TcSERPIN        AATCTTTCATTGAAGTTAATGAAGAAGGGACAGAAGCTGCAGCTGCTTCTGCTGGTGTTA
                ********** **.******************************  *:****** *.**.

Pseudogene      TCGTGGCATTTGGAAGTGCTACGTTTAGTTTAGGATACGATAGATTTCGTGGCTGACCAT
TcSERPIN        T-AAGACTCAGGGGTGTGCT------TGTTGAGGAAAAAAATAGATTTTGTGGCTGACCAT
                * .:*.*: : **.:*****      :*** ****:*..******** ************

Pseudogene      CCATTCCTTTTCATGATCACAGAGGATGTAAGTGGAGTTCTGCTGTTCATCGGGCATGTG
TcSERPIN        CCATTCCTCTTCCTGATCAGGGAAGATGTGACTGGAGTTGTTCTGTTCATTGGGCACGTC
                ******** ***.****** .**.*****.* ******* * ******** ***** **

Pseudogene      CTCAATCCCCTTTGAAAGCTGATAAATTTGCTAT---------AAAGGAGAATGAA--CA
TcSERPIN        CTCAATCCTCTTG-AAAGTTGATAAATCTGTTCTGATCCTTATGTAGGAAAATGTGTACG
                ******** ***  **** ******** ** *.*         .:****.****:.  *.

Pseudogene      ------TACCCCAGCATATCAAAGCCTGACAAGCAAATGATTCAGGATTGTGGAAATCTC
TcSERPIN        AGGAGGATAACTAGCTGAA--------------CAAATG--GAAGATTTGTGGTTATTTT
                      ::..* ***: *:               ******   .**.:******::** *

Pseudogene      AAGTATCTGGATGTTG-----CATTTCACACATGCCAAAGCATAGTATA-----------
TcSERPIN        AAGCATCCGGTTGATGTGTTGTAGTTTGCATATGCCAGAGCCTAGTGCCGCTTAAGTGGT
                *** *** **:**:**      * ** .** ******.***.****. .

Pseudogene      -------------------------TGCTTTCTGGTA-GCTTCGTTTTCTTTCGTTCTTT
TcSERPIN        TGTGTAGTCTAACCCGTGTGACTTATGCTTTCTTGTAAGTTTCCTTTT----CGTTTCC-
                                         ******** *** * *** ****    ****

Pseudogene      TTAATGTTTT---TGTTCTTCACTGCTAA-------------------GTTGTT--TAGT
TcSERPIN        --TATGTCTTTGCTGTTGATCCCAGTAGGTTATAACAATAAACTACTTGTTGTTCCCATT
                  :**** **   **** :**.*:* :..                   ******   * *

Pseudogene      TGTAAAGCAATCACATTGGAGTTGTA----------------------------------
TcSERPIN        AGAAATACATTCTCATGGCTTTTGACTTGTTGAATACTTAAAGCTTAGCTCTCCTCTCTT
                :*:**:.**:**:*** * : ***:.

Pseudogene      -
TcSERPIN        A
```

Supplementary Figure 1. Alignment of the gene regions of *TcSERPIN* and flanking pseudogenes at 3' and 5'UTR.
