# Supplementary material for: TcSERPIN, an inhibitor that interacts with cocoa defense proteins and has biotechnological potential against human pathogens
Source: Front Plant Sci. 2024 Jan 29;15:1337750. doi: 10.3389/fpls.2024.1337750 (PMC10859438; doi:10.3389/fpls.2024.1337750)
Supplement: Supplementary file 1 [file DataSheet_1.zip › Supplementary Figure 2.pdf]

```
TcSERPIN    -QTDVTLSLTKHVLQ-TE---AKDSNLAFSPLSIHVVLSMIAAGSTGPTLDQLLSFLKS---ASN
AtSerpin1   -QNQVSMNLAKHVIT-TV---SQNSNVIFSPASINVVLSIIAAGSAGATKDQILSFLKF---SST
CmPS-1      ---DVAMAITKRILQHDE---AKGSNVVISPLSIYVLLSLVAAGSKGRPLDQLLSFLKS---NSI
ZXA         HQTSFALRLAAALSSPAHPAGGAGRNVAFSPLSLHVALSLVAAGAGGATRDQLASALGG--PGSA
BSZx        HQTRFAVRLASAISSPSHAKGSS-GNAAFSPLSLHVALSLVAAGAA-ATRDQLAATLGAAEKGDA
WSZ2A       HQTRFGFRLASTISSNPE---STANNVAFSPVSLHVALSLITAGAGGATRDQLVATLGE---GEA

TcSERPIN    DHLGSFSSELVSVVFADGSPAGGPRLSFANGVWIDKSLPLKPSFKQVVDNVYKAASNQVDFQTKA
AtSerpin1   DQLNSFSSEIVSAVLADGSANGGPKLSVANGAWIDKSLSFKPSFKQLLEDSYKAASNQADFQSKA
CmPS-1      DNLNAFASHIIDKVFADASSCGGPRLAFVNGVWIDQSLSLKSSFQQVVDKYYKAELRQVDFLTKA
ZXA         EGLHAFAEQLVQLVLADASGAGGPRVAFADGVFVDASLSLKKTFGDVAVGKYKAETHSVDFQTKA
BSZx        EGLHALAEQVVQVVLADASGAGGPRSF--ANVFVDSSLKLKPSFKDLVVGKYKGETQSVDFQTKA
WSZ2A       ERLHALAEQVVQFVLADASYADSPRVTFANGVFVDASLPLKPSFQELAVCKYKAEAQSVDFQTKA

TcSERPIN    VQVAGEVNLWAEKETSGLIKQLLPPGSVDGSTRLIFANALYFKGAWNETFDASKTKENDFYLVNG
AtSerpin1   VEVIAEVNSWAEKETNGLITEVLPEGSADSMTKLIFANALYFKGTWNEKFDESLTQEGEFHLLDG
CmPS-1      NEVISEVNSWVEKNTYGLIREILPAGSVGSSTQLVLANALYFKAAWQQAFDASITMKRDFYLIDG
ZXA         AEVASQVNSWVEKVTSGLIKEILPPGSVDHTTRLVLGNALYFKGAWTEKFDASKTKDGEFHLLDG
BSZx        PEVAGQVNSWVEKITTGLIKEILPAGSVDSTTRLVLGNALYFKGSWTEKFDASKTKDEKFHLLDG
WSZ2A       AEVTAQVNSWVEKVTTGLIKDILPAGSISNTTRLVLGNALYFKGAWTDQFDSRVTKSDYFYLLDG

TcSERPIN    SSVKAPFMTSQKKQAVGAYDGFKVLGLPYKQGGDKRRFSMYFFLPDAKDGLPALVEKVSSESGFL
AtSerpin1   NKVTAPFMTSKKKQYVSAYDGFKVLGLPYLQGQDKRQFSMYFYLPDANNGLSDLLDKIVSTPGFL
CmPS-1      SSVKAPFMSGEKDQYVAVFDGFKVLALPYSQGPDPRRFSMYFFLPDRKDGLASLIEKLDSEPGFI
ZXA         KSVQAPFMSTSKKQYILSYDNLKVLKLPYQQGGDKRQFSMYILLPEAQDGLWSLAEKLNSEPEFL
BSZx        SSVQTPFMSSTKKQYISSYDSLKVLKLPYQQGGDKRQFSMYILLPEAQDGLWNLANKLSTEPEFM
WSZ2A       SSIQTPFMYSSEEQYISSSDGLKVLKLPYKQGGDKRQFSMYILLPEAPSGIWSLAEKLSAEPELL

TcSERPIN    ERHLPYEPVKVGEFRIPRFKISFGFEASEVLKRLGLVLPFSGEGGLTEMVDSP-LGQSLYVSNIF
AtSerpin1   DNHIPRRQVKVREFKIPKFKFSFGFDASNVLKGLGLTSPFSGEEGLTEMVESPEMGKNLCVSNIF
CmPS-1      DRHIPCKKQELGGFLIPKFKISFGIEVSDVLKKLGLVLPFT-EGGLLGMVESP-VAQNLRVSNIF
ZXA         EKHIPTRQVTVGQFKLPKFKISFGFEASDLLKSLGLHLPFSSEADLTEMVDSP-EGKNLFVSSVF
BSZx        EKHMPMQKVPVGQFKLPKFKISFGFEASDMLKGLGLQLPFSSEADLSEMVDSP-AARSLYVSSVF
WSZ2A       ERHIPRQKVALRQFKLPKFKISFGIEASDLLKHLGLQLPFSDEADLSEMVDSP-MPQGLRISSVF

                         P15 ------------> P1-P1'
TcSERPIN    HKSFIEVNEEGTEAAAASAGVIRLRGVLVE-EKIDFVADHPFLFLIREDVTGVVLFIGHVLNP
AtSerpin1   HKACIEVNEEGTEAAAASAGVIKLRGLLMEEDEIDFVADHPFLLVVTENITGVVLFIGQVVDP
CmPS-1      HKAFIEVDEEGTKAAASSAVTVGIVS--LPINRIDFIANRPFLYLIREDKSGTLLFIGQVLNP
ZXA         HKSFVEVNEEGTEAAAATAAVITLRSAP---IAEDFVADHPFLFLIQEDMTGVVLFVGHVVNP
BSZx        HKSFVEVNEEGTEAAARTARVVTLRSLPVEPVKVDFVADHPFLFLIREDLTGVVLFVGHVFNP
WSZ2A       HKTFVEVNETGTEAAAATIAKAVLLS-ASPPSDMDFIADHPFLFLIREDTSGVVLFIGHVVNP
                                   RCL
```

Supplementary Figure 2. Analysis of the primary structure of the TcSERPIN protein. Sequence alignment of amino acid residues of the domain of *Theobroma cacao* (TcSERPIN, Tc02v2_p008580.1), *Arabidopsis thaliana* (AtSerpin1, NP_190108.1), *Curcubita maxima* (CmPS-1, AAG02411.1), *Oryza sativa* (ZXA, XP_015632921.2), *Hordeum vulgare* (BSZx, Q40066.1), and *Triticum aestivum* (SPZ2A, Q9ST57.1). Conserved regions are highlighted in gray and gap regions are indicated by (-). The P_{15} – $P_{1'}$ region of the RCL is underlined. Phosphorylation and glycosylation sites are marked by rectangles and circles, respectively.
