# Supplementary material for: TcSERPIN, an inhibitor that interacts with cocoa defense proteins and has biotechnological potential against human pathogens
Source: Front Plant Sci. 2024 Jan 29;15:1337750. doi: 10.3389/fpls.2024.1337750 (PMC10859438; doi:10.3389/fpls.2024.1337750)
Supplement: Supplementary file 1 [file DataSheet_1.zip › Supplementary Figure 3.pdf]

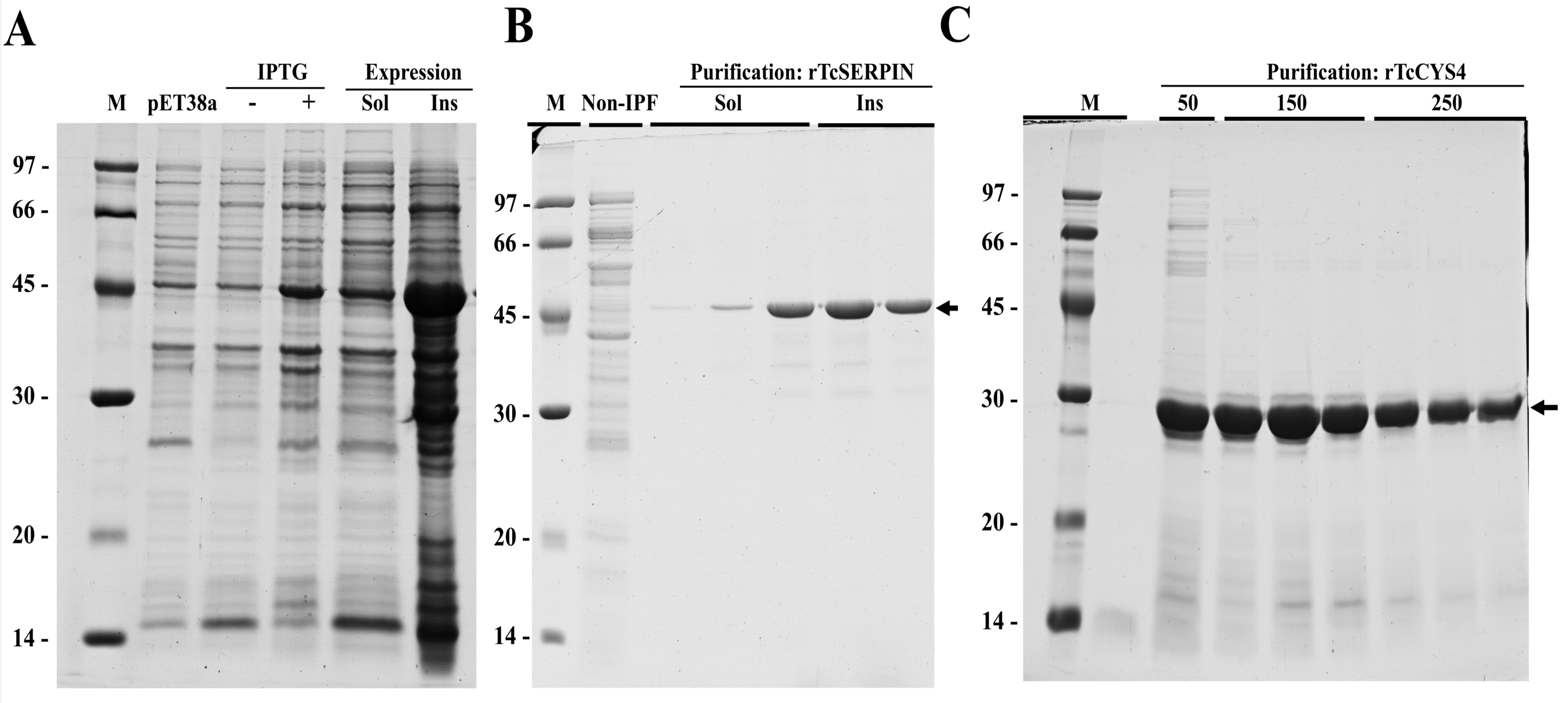

Supplementary Figure 3. Analysis of the expression, solubility and purification of recombinant cocoa inhibitors. **A**, SDS-PAGE of the expression of recombinant TcSERPIN in *E. coli*: M, molecular weight marker; pET28a; (-) and (+), induction of rTcSERPIN expression, without and with IPTG, respectively; Sol and Ins, expression of rTcSERPIN in the soluble and insoluble fractions of the bacterial extract. **B**, Result of the purification of rTcSERPIN from the soluble and insoluble fractions of the bacterial protein extract. **C**, SDS-PAGE of the purification of rTcCYS4 from the soluble fraction of the bacterial protein extract. The rTcCYS4 protein was eluted in the column at different concentrations of imidazole (50, 150 and 250mM). The black arrows mark the molecular weight of the purified proteins.
