# Supplementary material for: TcSERPIN, an inhibitor that interacts with cocoa defense proteins and has biotechnological potential against human pathogens
Source: Front Plant Sci. 2024 Jan 29;15:1337750. doi: 10.3389/fpls.2024.1337750 (PMC10859438; doi:10.3389/fpls.2024.1337750)
Supplement: Supplementary file 1 [file DataSheet_1.zip › Supplementary Figure 4.pdf]

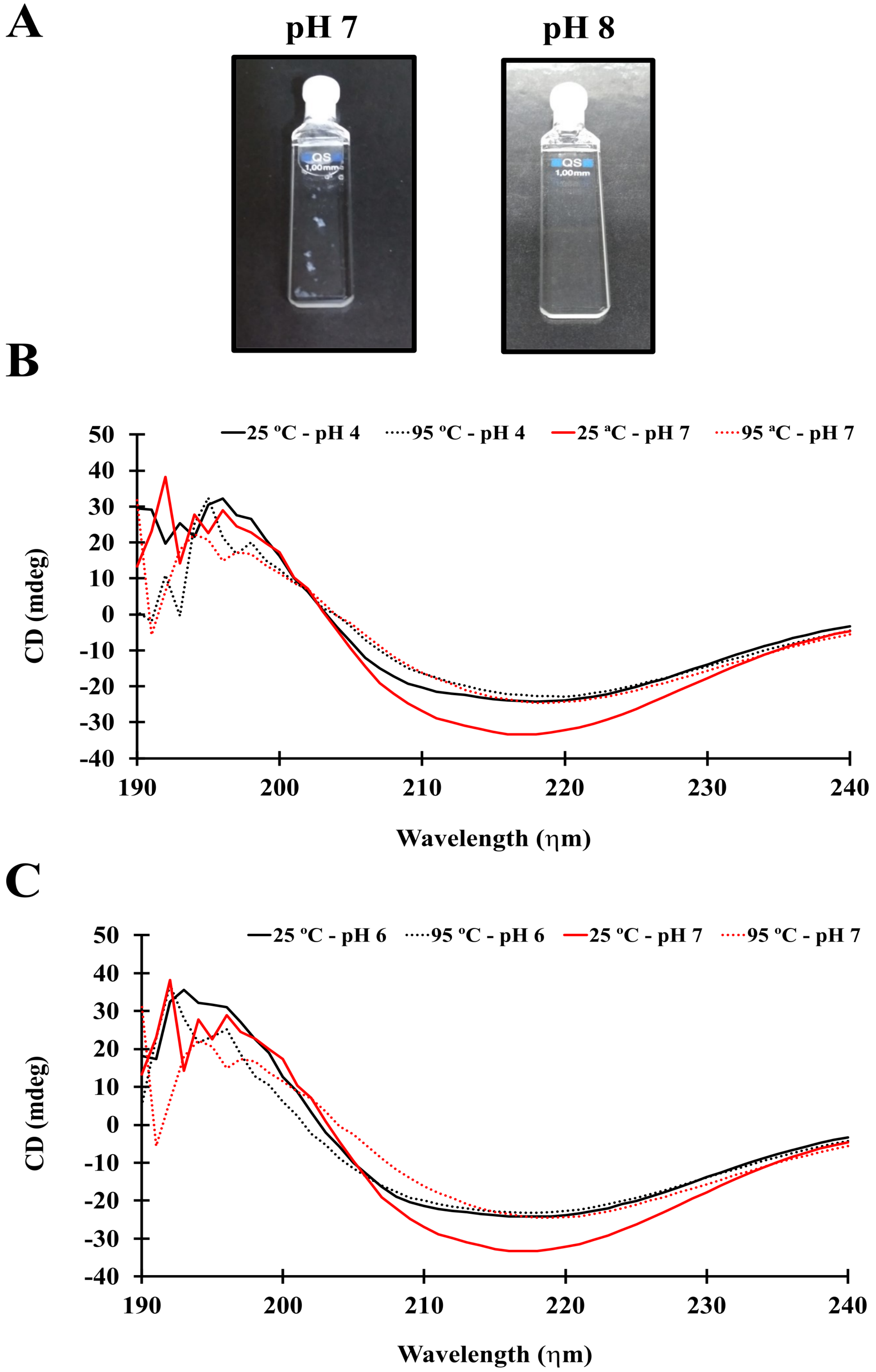

Supplementary Figure 4. Circular dichroism (CD) analysis of rTcSERPIN. **A**, Image of cuvettes after heating the rTcSERPIN protein to 95 ºC at pH 7 and 8, respectively. **B** and **C**, Spectral profiles by circular dichroism at wavelengths from 190 – 240 nm of the rTcSERPIN at pH 7 (red lines), 6 and 4 (black lines) treated at 25 °C (solid lines) and 95 °C (dashed lines). The spectrum of the protein analyzed at pH 4 and 6 was obtained in citrate buffer (10 mM), and for pH 7 it was obtained in Tris HCl (10 mM).
