# Supplementary material for: TcSERPIN, an inhibitor that interacts with cocoa defense proteins and has biotechnological potential against human pathogens
Source: Front Plant Sci. 2024 Jan 29;15:1337750. doi: 10.3389/fpls.2024.1337750 (PMC10859438; doi:10.3389/fpls.2024.1337750)
Supplement: Supplementary file 1 [file DataSheet_1.zip › Supplementary Figure 5.pdf]

```
P43297_Cysteine_proteinase_RD21A_Arabidopsis    MGFLKPTMAILFLAMVAVSSAVDMSIISYDEKHGVST-TGGRSEAEVMSIYEAWLVKHGK
A0A061FBU0_Cysteine_protease_Theobroma          MGSQRSTMAMLLLVMFTLSSALDMSIISYDEGHPDKSKSIWRTDDEVMAMYEEWLVKHGK

P43297_Cysteine_proteinase_RD21A_Arabidopsis    AQSQNSLVEKDRRFEIFKDNLRFVDEHNEK-NLSYRLGLTRFADLTNDEYRSKYLGAKME
A0A061FBU0_Cysteine_protease_Theobroma          AY--NGLGEKERRFEIFKDNLRFIDEHNADDSHSFKVGLNRFADLTNEEYRAMYLGTKKP

P43297_Cysteine_proteinase_RD21A_Arabidopsis    KKGERRTSLRYEARVGDELPESIDWRKKGAVAEVKDQGGCGSCWAFSTIGAVEGINQIVT
A0A061FBU0_Cysteine_protease_Theobroma          ERKVSKRSDRYAPSLGEELPDSIDWREKGAVAAVKDQGGCGSCWAFSAIAAVEGINKIVT

P43297_Cysteine_proteinase_RD21A_Arabidopsis    GDLITLSEQELVDCDTSYNEGCNGGLMDYAFEFIIKNGGIDTDKDYPYKGVDGTCDQIRK
A0A061FBU0_Cysteine_protease_Theobroma          GDLIVLSEQELVDCDTTYNEGCNGGLMDYAFEFIINNGGIDTEEDYPYTGRDGTCDPYRK

P43297_Cysteine_proteinase_RD21A_Arabidopsis    NAKVVTIDSYEDVPTYSEESLKKAVAHQPISIAIEAGGRAFQLYDSGIFDGSCGTQLDHG
A0A061FBU0_Cysteine_protease_Theobroma          NARVVSIDAYEDVPVNDETALKKAVANQPVSVAIEAGGRAFQLYQSGIFDGKCGTQLDHG

P43297_Cysteine_proteinase_RD21A_Arabidopsis    VVAVGYGTENGKDYWIVRNSWGKSWGESGYLRMARNIA-SSSGKCGIAIEPSYPIKNGEN
A0A061FBU0_Cysteine_protease_Theobroma          VTAVGYGTEKGKDYWIVKNSWGSSWGEEGYIRMARNEANSVTGKCGIAIEASYPIKKGQN

P43297_Cysteine_proteinase_RD21A_Arabidopsis    PPNPGPSPPSPIKPPTQCDSYYTCPESNTCCCLFEYGKYCFAWGCCPLEAATCCDDNYSC
A0A061FBU0_Cysteine_protease_Theobroma          PPNPGPSPPSPIKPPTVCDSYYTCPESNTCCCVYEYYGYCFAWGCCPLEAATCCDDHYSC

P43297_Cysteine_proteinase_RD21A_Arabidopsis    CPHEYPVCDLDQGTCLLSKNSPFSVKALKRKPATPFWSQGRKNIA---
A0A061FBU0_Cysteine_protease_Theobroma          CPHEYPICNINEGTCLMSKGNPLGVKALRRTPAKPFWAHGSVGKKSNA
```

Supplementary Figure 5. The alignment was carried out with the amino acid residue sequences of the cysteine proteases from *Theobroma cacao* and *Arabidopsis thaliana* identified by mass spectrometry and interaction network analysis (PPI), respectively. According to the alignment, proteases have 73% identity with each other. The C1 domain of proteases appears underlined with a solid black line, and the granulin domain located in the C-Terminal region is marked with a dashed line.
